# Measuring habitat quality for waterbirds: A review

**DOI:** 10.1002/ece3.9905

**Published:** 2023-04-07

**Authors:** Rowan Mott, Thomas A. A. Prowse, Micha V. Jackson, Daniel J. Rogers, Jody A. O'Connor, Justin D. Brookes, Phillip Cassey

**Affiliations:** ^1^ School of Biological Sciences The University of Adelaide Adelaide South Australia Australia; ^2^ Department for Environment and Water Adelaide South Australia Australia

**Keywords:** environmental proxies, habitat condition, habitat selection, population growth rate, wetland monitoring

## Abstract

Quantifying habitat quality is dependent on measuring a site's relative contribution to population growth rate. This is challenging for studies of waterbirds, whose high mobility can decouple demographic rates from local habitat conditions and make sustained monitoring of individuals near‐impossible. To overcome these challenges, biologists have used many direct and indirect proxies of waterbird habitat quality. However, consensus on what methods are most appropriate for a given scenario is lacking. We undertook a structured literature review of the methods used to quantify waterbird habitat quality, and provide a synthesis of the context‐dependent strengths and limitations of those methods. Our search of the Web of Science and Scopus databases returned a sample of 666 studies, upon which our review was based. The reviewed studies assessed habitat quality by either measuring habitat attributes (e.g., food abundance, water quality, vegetation structure), or measuring attributes of the waterbirds themselves (e.g., demographic parameters, body condition, behavior, distribution). Measuring habitat attributes, although they are only indirectly related to demographic rates, has the advantage of being unaffected by waterbird behavioral stochasticity. Conversely, waterbird‐derived measures (e.g., body condition, peck rates) may be more directly related to demographic rates than habitat variables, but may be subject to greater stochastic variation (e.g., behavioral change due to presence of conspecifics). Therefore, caution is needed to ensure that the measured variable does influence waterbird demographic rates. This assumption was usually based on ecological theory rather than empirical evidence. Our review highlighted that there is no single best, universally applicable method to quantify waterbird habitat quality. Individual project specifics (e.g., time frame, spatial scale, funding) will influence the choice of variables measured. Where possible, practitioners should measure variables most directly related to demographic rates. Generally, measuring multiple variables yields a better chance of accurately capturing the relationship between habitat characteristics and demographic rates.

## Graphical abstract

Our review of studies that measured waterbird habitat quality found that practitioners typically use proxies for habitat quality that focus on some aspect of the habitat or some aspect of the waterbirds themselves. The former may be relatively insensitive to waterbird behavioral stochasticity, whereas the latter may be more directly linked to waterbird demographic rates. We recommend that practitioners base their choice of variable to measure on the ecology of their study species, and preferentially measure variables most directly linked to waterbird demographic rates.

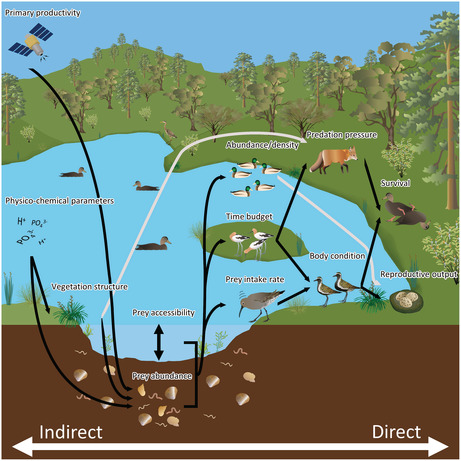

## 1 INTRODUCTION

A core aim of conservation management is optimizing habitat quality for focal species (Johnson, 2005; McComb, 2016). For management to be truly optimized, a measurable understanding of what constitutes habitat quality is required (Marzluff et al., 2000). The term habitat has diverse meanings in the literature. For the purposes of this review, we follow the definition provided by Kearney (2006) whereby habitat is a spatially and temporally explicit description of the physical features, including abiotic and biotic elements, of a place where an organism either actually or potentially lives. The ultimate measure of the quality of a habitat for an individual is the individual's relative contribution to the growth rate of the population when inhabiting a given habitat (Johnson, 2007). There are two components of this measure: survival and reproduction. By defining habitat quality in terms of population growth rate, habitat quality can be assessed on a continuous temporal scale. For example, habitat quality can be measured instantaneously or as a life‐time measure of habitat quality akin to the individual's fitness. There are many components that combine to influence survival and reproductive output including food availability, predation risk, habitat structure and configuration, and the presence of disturbances (e.g., human foot traffic; Johnson, 2007). Moreover, different species will experience habitat in different ways because of the species‐specific mechanistic ways in which they interact with features of the habitat (i.e., their respective niches *as per* the definition of Kearney (2006)) and habitat quality is therefore species‐specific.

Quantifying demographic rates (survival and reproductive output) is a challenging task (Stephens et al., 2015), as it requires sustained monitoring of individuals of known identity. Studies that do achieve this are often conducted either on sessile organisms (e.g., Ma et al., 2014; Wang et al., 2012; Zhao et al., 2006) or large‐bodied organisms that are restricted to a small geographic area (e.g., islands: Kruuk et al., 1999, Richard et al., 2014; natal colony: Baker & Thompson, 2007; Le Boeuf et al., 2019). Demographic rates are also financially costly to measure (Knutson et al., 2006; Pidgeon et al., 2006), and the long timeframes for data collection can mean that research extends beyond typical funding cycles and research project lifetimes, particularly for research on long‐lived species (Le Boeuf et al., 2019). Despite these challenges, there have been studies that successfully monitor survival (Valdez‐Juarez et al., 2019) and reproductive performance (Pérot & Villard, 2009; Pidgeon et al., 2006; Zanette, 2001) of birds in relation to habitat quality. Outputs from these studies are often very applied with actionable recommendations for conservation decision‐makers.

Waterbirds are a particularly challenging group to obtain habitat quality estimates for because multiple factors can confound the relationship between site habitat conditions and resultant demographic rates. The distribution of many waterbird species is influenced by social attraction (Gawlik & Crozier, 2007). As a result, areas of favorable habitat may go unused because waterbirds newly arriving in an area are drawn to sites with existing waterbird presence (Gawlik & Crozier, 2007). Moreover, many waterbirds are highly dispersive and track ephemeral habitat conditions at local, regional, or even continental scales (Cumming et al., 2012; Pedler et al., 2014; Roshier et al., 2006), creating the potential for mismatches between the scale of monitoring and the scale at which demographic processes are governed.

Dispersive behavior can also mean that the conditions at a particular wetland may be conducive for survival and reproduction, yet waterbirds do not capitalize on these favorable conditions because other wetlands in the broader landscape are also providing favorable conditions (behavioral choice impacts) (Cumming et al., 2012). This poses a challenge for management because under the theoretical definition of habitat quality, unused sites are not conferring anything to an individual's relative contribution to population growth rate and so the wetland would be deemed low‐quality habitat. Yet, from a management perspective, the site is being managed to provide conditions consistent with high‐quality habitat. Similarly, this scenario also highlights the role of density in habitat quality when habitat quality is assessed at the level of the population rather than the individual. Although a site may support only modest population growth from an individual's perspective, if the site has a high population density, then the population growth rate may be relatively high. Conversely, if a site has only a small number of resources but these are high‐quality resources (e.g., optimum nest sites), the per capita contribution to population growth rate at the site may be high, but the site has low habitat quality from a population perspective because the density of individuals supported by the site is low.

Many waterbirds are also migratory. Consequently, demographic parameters in one part of the range may be decoupled from the habitat conditions experienced at that time due to carry‐over effects from previous seasons (Aharon‐Rotman, Bauer, & Klaassen, 2016; Sedinger & Alisauskas, 2014; Swift et al., 2020). For example, survival during the breeding period and breeding success may be higher in individuals that depart their nonbreeding grounds in better condition (Swift et al., 2020). Furthermore, breeding performance in one part of the range may influence parameters including abundance and population age structure on the nonbreeding grounds, irrespective of the local conditions on the nonbreeding grounds (Rogers & Gosbell, 2006). In addition to carryover effects, survival data may be particularly sensitive to pinch points of low‐quality habitat along the migratory flyway (Piersma et al., 2016; Studds et al., 2017).

Due to the difficulties of obtaining waterbird demographic data in a given area, an array of methods have been used as proxies to measure habitat quality (Ma et al., 2010). The use of proxies also helps to overcome budget limitations of management agencies by allowing snapshot estimates of habitat quality to be made without the need for extended periods of data collection in space and time (Osborn et al., 2017). However, the many different options available for measuring habitat quality can be bewildering for research scientists and conservation practitioners (Pidgeon et al., 2006). There is little consensus on which method, or combination of methods, produces the most meaningful estimate of waterbird habitat quality, and in some cases, it is unclear as to whether particular proxies meaningfully reflect underlying habitat quality from the perspective of direct impact on population processes (Johnson, 2005, 2007; Stephens et al., 2015; Van Horne, 1983). For example, density of individuals may not reflect underlying habitat quality if the population does not follow the ideal free distribution (Van Horne, 1983), and time spent foraging may not reflect underlying habitat quality if individuals are constrained by prey handling time or digestive bottlenecks (Van Gils et al., 2005). Furthermore, the spatial scale at which proxies are measured may have implications for their relevance to managers (Guisan & Thuiller, 2005; Pidgeon et al., 2006; Stephens et al., 2015). The dynamic nature of wildlife populations also means that meta‐population processes (e.g., local extinction and recolonization, source‐sink dynamics), including those of competitors and predators, may confound proxy measures for habitat quality because these proxies typically assume the study population is in equilibrium (Guisan & Thuiller, 2005; Pulliam, 2000).

In this review, we seek to catalogue the methods that have been used to quantify waterbird habitat quality and provide a synthesis of the conditions under which each may provide meaningful measures of habitat quality in future waterbird studies. Outputs from this review are intended to guide environmental managers on the types of data they should be collecting when attempting to quantify waterbird habitat quality. This will ensure that decisions on how to manage habitat to optimize habitat quality are based on meaningful information. Outputs are also expected to be useful for conservation practitioners in terrestrial settings because the factors governing the relationship between a proxy and underlying habitat quality are founded on general ecological principles. Therefore, support for a method in a waterbird context is likely to mean that the same data type would provide a useful habitat quality proxy in a terrestrial setting. Furthermore, the open habitat structure occupied by many waterbird species (e.g., bare mudflats) facilitates prolonged periods of observation of individuals relative to those from terrestrial settings such as forests and scrublands. Likewise, visual methods for assessing body condition have been developed for waterbirds (Féret et al., 2005; Wiersma & Piersma, 1995), whereas capture is required to collect the same data for terrestrial birds (e.g., Milenkaya et al., 2015). Therefore, waterbirds provide a novel test‐case to quantify the relationship between some purported habitat quality proxies and the expected outcome (e.g., influence of body condition on survival and reproduction) where evidence from terrestrial settings remains unclear (Barnett et al., 2015; Milenkaya et al., 2015).

## 2 METHODS

For the purposes of this review, we followed the definition of waterbirds used by Wetlands International (2012). This covers all species within 32 bird families that are ecologically dependent on wetlands. The most familiar of these families are the Anatidae (ducks, geese, and swans), Laridae (gulls and terns), Ardeidae (herons and egrets), Scolopacidae (sandpipers), and Charadriidae (plovers). Other representatives include the Rallidae (rails and crakes), Podicipedidae (grebes), Threskiornithidae (ibises and spoonbills), and Recurvirostridae (stilts and avocets).

Systematic reviews require defining the question elements ‘subject’, ‘intervention’, ‘outcome’, and ‘comparator’ (Pullin & Stewart, 2006). Very few studies of habitat quality involve manipulative experiments (Stephens et al., 2015), so for the majority of studies there is a lack of a clearly defined intervention. Furthermore, for data to be pooled for meta‐analysis, it is important to consider the heterogeneity of the input data, with high levels of heterogeneity making data pooling inappropriate (Crowther et al., 2010). For example, it is important that only data with similar interventions and measures of outcomes are combined (Crowther et al., 2010). Among the diverse waterbird habitat quality literature, there are clear differences in terms of the experimental design, comparator group used (e.g., some use neighboring populations as comparators whereas others use previous points in time, or a comparator is lacking completely), and the measured outcome (e.g., some relate changes in a measured variable to change in abundance whereas for others the response is changes in reproductive rate). Consequently, pooling data to conduct a formal meta‐analysis would have been inappropriate in this review. Hence, we used the ‘narrative synthesis’ approach recommended by Haddaway et al. (2020) for synthesizing heterogeneous literature. To obtain a representative sample of the literature for synthesis, we used a structured approach to identify relevant information sources (published literature, reports, and gray literature) and use these sources to make qualitative assessments of the various methods that have been used for measuring waterbird habitat quality.

We searched the Web of Science (all databases) and Scopus on September 1, 2022 to obtain a set of papers on which to base this review. When searching the Web of Science we used the search string TS = (waterbird* OR shorebird* OR wader* OR “wading bird*” OR waterfowl) AND TS = (“habitat quality” OR “habitat condition” OR “environment* quality” OR “environment* condition” OR “wetland quality” OR “wetland condition”), where TS means ‘Topic Search’.

When searching the Scopus database we specified two search fields. The first contained the search string waterbird* OR shorebird* OR wader* OR “wading bird*” OR waterfowl and the second contained the search string “habitat quality” OR “habitat condition” OR “environment* quality” OR “environment* condition” OR “wetland quality” OR “wetland condition”. In both cases, the search was restricted to include only information appearing in the article title, abstract, keywords.

These searches returned a sample of 1085 results once duplicates and records pertaining to datasets (e.g., publication supporting information lodged with Dryad or Figshare) had been removed. Further screening removed an additional 158 papers that did not relate to waterbirds, 155 papers in which no habitat quality assessment was made, 25 studies that related to toxicology, and 52 review articles. It was not possible to obtain a copy of the full text in 29 cases and these records were also excluded from the synthesis. This resulted in a final sample of 666 papers upon which our synthesis was based (see Appendix S1 for a spreadsheet of search results and inclusion status). For cases where the returned paper was in a language other than English or French, Google Translate was used to extract the relevant information. Our aim in conducting this review was to catalogue the spectrum of methods that have been used for waterbird habitat quality assessments. Therefore, we did not appraise the quality of individual studies when determining whether they should be included in the review or not. Rather we included any study that purported to measure habitat quality and used evidence from within that study or others in the returned set to contextualize whether the method was likely to provide an accurate estimate of habitat quality, as well as outlining any caveats as to the settings under which the method is likely to provide useful data. It was intended that this would help readers make their own assessment as to whether a particular habitat quality metric is useful for their own needs. We also avoid ‘vote counting’ (i.e., tallying how many studies used a particular method) as a way of assessing how well a given habitat quality assessment method performs because there are many factors that can contribute to a particular method being used (e.g., ease of data collection), or the likelihood of the research being published (e.g., sample size needed for statistical power) that are unrelated to the performance of that method.

## 3 SYNTHESIS OF REVIEWED STUDIES

Our structured search returned studies that undertook waterbird habitat quality assessments in two main ways: studies that measured some biophysical attribute(s) of the habitat; and studies that measured some attribute(s) of waterbirds themselves to infer underlying habitat quality (Table 1). Studies that measured attributes of waterbirds themselves could be further broken down into four subcategories: studies that directly measured waterbird demographic characteristics; studies that measured waterbird body condition; studies that measured waterbird behavior; and studies that measured waterbird distribution (Table 1). There were also studies that used methods from a combination of these categories.

**TABLE 1:** Catalogue of methods used to assess waterbird habitat quality in studies reviewed as part of the structured literature review. For each method, examples of studies that used the method are given along with an indication of the support or lack thereof for the given method.

Method	Metrics	Supporting evidence	Contradictory evidence	Relevant spatial and temporal scales
Direct habitat measures				
Food availability	Prey animal biomass (Atiénzar et al., 2012; Deboelpaep et al., 2020; Herring & Gawlik, 2013; Holopainen et al., 2014; Hunt et al., 2017; Parks et al., 2016; Schultz et al., 2020) Plant‐derived food density or abundance (Arzel et al., 2015; Atiénzar et al., 2012; Dugger & Feddersen, 2009)	Birds behaviourally track sites with highest prey biomass and density (Rose & Nol, 2010), and if only accessible prey can be measured then this relationship can be even stronger than if overall biomass is measured (Lourenço et al., 2005) Prey availability has a positive influence on reproductive performance (Herring et al., 2010) Chick condition is related to local prey abundance (Hunt et al., 2017; Le Fer et al., 2008)	Predicts occupancy but not abundance (Gillespie & Fontaine, 2017) Sites with high food densities are not always the favored foraging sites (Hagy & Kaminski, 2015) and this can be due to accessibility (e.g., vegetation structure (Nam et al., 2015) or substrate hardness (Nolet & Klaassen, 2009)) The seeds of different plant species consumed by waterfowl have different energy content (Dugger et al., 2007) Different food items can result in different mass gain even when fed ad libitum (Jorde et al., 1995) Waterbirds may forage selectively on larger size‐class prey items meaning that overall prey density is not reduced through waterbird foraging even though waterbirds' preferred prey size has been significantly depleted (Fonseca & Navedo, 2020). Similarly, the availability of energy rich prey may be a more important factor determining reproductive success than total prey availability (Rizzolo et al., 2015) Social cues may override selection for habitat with the highest prey density (Master et al., 2005) Handling time may result in an asymptotic curve for intake rate at high food density (Smart & Gill, 2003) There is a prey biomass threshold below which it becomes unprofitable for waterbirds to continue foraging. This ‘give up density’ of prey should be, but rarely is, excluded from carrying capacity estimations (Mu et al., 2022)	Site/region—instantaneous/within season/annual
Primary productivity	Normalized Difference Vegetation Index (NDVI) (Tang et al., 2016; Zhang et al., 2017) Enhanced Vegetation Index (EVI) (Guan et al., 2016)	Landscape level parameters such as primary productivity were more important determinants of nest success than local‐scale variables (e.g., vegetation structure around the nest) most likely as a result of their influence on predator populations (Ringelman et al., 2018)	The method provides an indirect indication of habitat quality with at least one further transitional state before primary productivity influences waterbird energy intake rate (Zhang et al., 2017)	Site/region/flyway—instantaneous/within season/annual
Predation pressure	Predator track density (Cohen et al., 2009) Index of predator reproduction (Trinder et al., 2009) Proportion of radio‐tracked individuals predated (Kenow et al., 2009; Swift et al., 2020) Proportion of real or fake nests predated (Pehlak & Lõhmus, 2008; Swift et al., 2020) Alternate prey density (Holopainen et al., 2014)	Predation can be the leading cause of waterbird nest failure (Riecke et al., 2019) Predation risk is evaluated by waterbirds and trade‐offs made that may reduce other components of fitness (e.g., foraging rate) (Austin et al., 2017; Fernández & Lank, 2010; Maslo et al., 2012; Zharikov et al., 2009) Nest predation rate increases with the abundance of avian nest predators (Manton & Angelstam, 2021), and conversely, predator control (mammal and avian) increased the percentage of pairs that successfully raised young (Fletcher et al., 2010)	Nest predation rate was not a function of predator abundance or the availability of alternate prey species (Machín et al., 2019) The influence of predation can differ depending of the waterbird population density (Lebeuf & Giroux, 2014) The abundance of alternate prey and the abundance of foxes did not affect reproductive success for the majority (16 out of 17) shorebird taxa (Weiser et al., 2018a), nor did they typically affect adult survival (Weiser et al., 2018b) High predator abundance may reduce the proportion of nests that are successful but not the number of chicks produced per nest. When individuals are able to renest, predator abundance may not be a limiting factor on population growth rate (Cohen et al., 2009)	Site/region—instantaneous/within season/annual
Vegetation structure	Vegetation height (Barati et al., 2011) Vegetation cover/abundance (Atiénzar et al., 2012; Hamza et al., 2015; Hierl et al., 2007; Nyman & Chabreck, 1996) Vegetation community composition (Benedict & Hepp, 2000; Dugger & Feddersen, 2009) Presence of invasive plants (Khan, 2010; Tavernia & Reed, 2012)	Vegetation structure has implications for the suitability of a site for nest placement (Barati et al., 2011; Koivula & Rönkä, 1998), and influences probability of a territory being occupied (Minias & Janiszewski, 2016) Tall vegetation surrounding a wetland may decrease survival through increased predation pressure as a result of predator concealment (Boyer et al., 2018)	Dense vegetation may increase prey abundance but reduce prey capture efficiency, but the effect is weak in relation to other factors such as water depth (Lantz et al., 2011)	Site/region—instantaneous/within season/annual
Wetland spatial attributes	Connectivity to neighboring wetlands (Sebastián‐González, Sánchez‐Zapata, & Botella, 2010) Pond area (Atiénzar et al., 2012; He et al., 2009; Merendino & Ankney, 1994) Shoreline irregularity (Merendino & Ankney, 1994)	Pond size and distance to the nearest neighboring wetland are important determinants of waterbird habitat selection (Sebastián‐González, Sánchez‐Zapata, & Botella, 2010)	Cycles of hydrological stress (drought/non‐drought) can influence waterfowl habitat preferences, with birds seeking relatively deeper water bodies during drought irrespective of other habitat variables that are influential in wet years (Atiénzar et al., 2012) Even closely related species may respond in different ways to the same wetland attribute (e.g., pond size) (Mascitti, 2001)	Site/region—instantaneous/within season/annual
Water level	Drawdown (Herring & Gawlik, 2013; Townsend et al., 2006) Water level variability (Collazo et al., 2002) Availability of shallow water (Collazo et al., 2002; Gawlik & Crozier, 2007; Lantz et al., 2011) or exposed mud habitats (Davis & Smith, 1998; Ge et al., 2007) Landscape depth heterogeneity (Beerens et al., 2015)	Wading birds preferentially selected ponds that had been experimentally manipulated to have shallow rather than deep water (Gawlik & Crozier, 2007) and waterbird species richness and density correlates with the availability of shallow water habitats (Wang & So, 2003) Shorebirds consistently selected shallow water wetlands despite changes in wetland availability meaning that this required shifting from temporary to seasonal wetlands (Steen et al., 2018) Water level recession rate was a key influence on physiological condition of two species of waterbirds (Herring & Gawlik, 2013) For species that breed on exposed mud flats, the availability of mud flats is the primary driver of breeding occurrence (Bucher & Curto, 2012)	Water level variability did not influence habitat selection of wading birds (Gawlik & Crozier, 2007), which may be due to habitat selection responding more strongly to social cues than physical habitat attributes (Master et al., 2005)	Site/region—instantaneous/within season/annual
Disturbance	Distance to footpaths, roads, or railways (Burton et al., 2002; Hu et al., 2016; Li et al., 2019) Human settlements (Li et al., 2019)	The presence of people and vehicles nearby (≤50 m) reduces foraging rates (Maslo et al., 2012) Likewise, time spent foraging and flock density were reduced at a highly disturbed site (Swift et al., 2020) Disturbance by human activity and farming rather than availability of foraging areas more strongly influences waterbird species richness and abundance (Quan et al., 2002)	Human activities (e.g., clam harvesting) may have positive effects on waterbirds, especially shorebirds (Hamza et al., 2015) Human activity can have no effect on reproductive output where overall levels of human activity are relatively low (Markkola & Karvonen, 2020) Human activity may have no effect or even be positively correlated with shorebird abundance if it is more profitable to remain in a location with abundant food resources than the costs of human disturbance (Neuman et al., 2008) Habitat selection may be influence more by long‐term patterns of disturbance than instantaneous levels of disturbance (Peters & Otis, 2007)	Site/region—instantaneous/within season/annual
Foraging substrate	Sediment grain size (Reurink et al., 2015; Rose & Nol, 2010) Organic carbon content (Hamza et al., 2015; Reurink et al., 2015) Mud content (Hamza et al., 2015)	Prey biomass is strongly predicted by physical environment conditions including organic content and particle sizes of the sediments (Lovvorn et al., 2018; Rose & Nol, 2010)	Biomass of prey items can change considerably over short spatial scales even though sediment characteristics do not change (Lourenço et al., 2005)	Site/region—instantaneous/within season/annual
Land use	Proportion of agricultural land use (Austin et al., 2001; Duncan et al., 1999) Mariculture (Li et al., 2019) Mining (Li et al., 2019)	Changing land use can cause ecological traps if agricultural landscapes appear similar to natural landscapes (e.g., grasslands) but offer lower habitat quality (Buderman et al., 2020)	Factors such as traditional site use by waterbirds can confound the signal of change in response to changing land use (Tombre et al., 2005)	Site/region—instantaneous/within season/annual
Water chemistry	Color/turbidity (Atiénzar et al., 2012; Merendino & Ankney, 1994) pH (Merendino & Ankney, 1994; Walsh et al., 2006) Conductivity/salinity (Atiénzar et al., 2012; Merendino & Ankney, 1994) Dissolved nutrients (Merendino & Ankney, 1994; Pöysä et al., 2001; Walsh et al., 2006) Chlorophyll‐α concentration (Atiénzar et al., 2012)	Prey biomass is influenced by salinity (Rose & Nol, 2010) Water chemistry variables including pH, salinity, and concentrations of phosphorous, nitrogen and potassium concentration can be a predictor of occurrence of breeding ducks and duckling growth rate (Sjöberg et al., 2000; Walsh et al., 2006)	The range of variation in some water quality parameters may be too small to influence habitat quality for waterbirds (i.e., it does not surpass thresholds that would cause a measurable effect) (Russell et al., 2014)	Site/region—instantaneous/within season/annual
Climate and weather	Temperature Precipitation Wind Snow/ice cover	Large‐scale climate indicators such as Palmer Drought Severity Index and North Atlantic Oscillation can be strong predictors of population demographic parameters, likely due to their influence on factors such as wetland availability and primary productivity (McKinnon et al., 2022; Roy et al., 2019) Cold temperatures can reduce digestive efficiency for chicks with limited thermoregulation ability, thereby reducing their capacity for growth even when food is available (Krijgsveld et al., 2003) Extreme rainfall events can reduce waterbird density (Ross et al., 2018) Cold air temperatures can increase waterbird physiological stress markers due to reduced food availability (Legagneux et al., 2013) Cold temperatures and rainfall can negatively influence nest success, especially when cold and rainy conditions co‐occur (Pierce et al., 2019) Probability of departing a site increases with adverse wind, temperature and rainfall conditions (Xu & Si, 2019)	Temperature can have contrasting effects depending on the stage of breeding (Chyb & Minias, 2022)	
Terrain features	Elevation (van de Pol, Ens, et al., 2010) Slope (Hazlitt, 2001) Aspect (Peters & Otis, 2007)	Shorebird nest sites on steeply sloping shorelines had lower hatching success, likely due to reduced food availability (Hazlitt, 2001) Waterbird abundance is often greater in wetlands with shallowly sloping and less undulating shorelines (Hsu et al., 2014; Neuman et al., 2008; Schaffer‐Smith et al., 2018) Elevation relative to shoreline modulates flooding risk (van de Pol, Ens, et al., 2010) Elevation can have an important influence on the probability of wetland occupancy at the regional scale (Xu et al., 2019; Zhang et al., 2018) Roost sites may be selected based on whether aspect provides shelter from wind (Peters & Otis, 2007)	Elevation may be unrelated to breeding pair density, but correlated with the number of chicks that fledge. Therefore, different measures of reproductive output may give contrasting results (Godfrey et al., 2003)	
Bird‐derived estimates				
Demographic measures				
Reproduction	Clutch size/volume (Hunt et al., 2017; Mallory et al., 1994; Powell & Powell, 1986) Number of fledglings (Powell & Powell, 1986)	Poor chick production can be directly responsible for a shift from population growth to decline because in situ recruitment is insufficient to sustain the local population despite high adult survival (Weegman et al., 2022)	Breeding success varies markedly in natural settings (Cleasby et al., 2017; Dickson, 1992; Reed et al., 2003) and therefore the effect of habitat change on reproductive performance must be large in order to detect the difference (Dickson, 1992) Indicators of reproductive performance early in a breeding cycle may be a poor indicator of eventual recruitment into the breeding population (Kwon et al., 2021; Smart et al., 2013)	Site/region—instantaneous/within season/annual
Survival	Adult survival (Alves et al., 2013; Rice et al., 2007; Swift et al., 2020) Brood survival (Aubry et al., 2013; Cohen et al., 2009; Hunt et al., 2017; Owen & Pierce, 2014; Simpson et al., 2007; Swift et al., 2020)	Adult survival can be the parameter most strongly influencing population growth rate (Cleasby et al., 2017)	Intrinsic buffers may mean that populations are able to withstand small differences in habitat quality (e.g., between population differences in migration costs or length of breeding season) without any impact of survival (Souchay et al., 2015)	Site/region—instantaneous/within season/annual
Density or abundance	Density (Loewenthal et al., 2015; Swift et al., 2020) Abundance (Castillo‐Guerrero et al., 2009; Dugger & Feddersen, 2009; Ganzevles & Bredenbeek, 2005; Hickman, 1994; Liu et al., 2006) Species richness (Dugger & Feddersen, 2009; Hickman, 1994) Abundance of breeding pairs (Arzel et al., 2015; Austin et al., 2001; Sebastián‐González, Botella, Sempere, & Sánchez‐Zapata, 2010)	The density of breeding pairs increased much faster than could be explained by population growth rates following habitat management that resulted in greater food availability (Loewenthal et al., 2015). This was attributed to previously subordinate adults taking up breeding territories as territory size of existing pairs contracted (Loewenthal et al., 2015)	Can be confounded by site fidelity (O'Neil et al., 2014), lags in response to change in condition (Loewenthal et al., 2015; Meltofte, 2006), dispersal barriers or costs, and imperfect knowledge of habitat (Lewis et al., 2010; Stillman et al., 2005) Local and regional weather influences habitat use (Kelly, 2001; Schummer et al., 2010) Reproductive output is not correlated with population density (Cohen et al., 2009; Holopainen et al., 2014) Reduction in food availability can increase shorebird density as they are concentrated into the remaining suitable patches (Kosztolányi et al., 2006) Requires birds to correctly perceive habitat cues, which may not always be the case (e.g., agricultural land uses may resemble native grasslands, but have much lower reproductive output) (Buderman et al., 2020) The influence of nest density on nest success can change throughout the season, initially being positive but declining as predators concentrate in areas with high nest density (Ringelman et al., 2018) Local changes in abundance may be the result of spill over from high‐quality sites if the overall population size is increasing rather than from changes in local habitat quality (Ntiamoa‐Baidu et al., 2014) Natural variability can mean that long‐term (decades) monitoring is required to detect even small reductions in abundance with statistical significance (Warnock et al., 1998)	Site/region—instantaneous/within season/annual
Age at first breeding	Age at first breeding (Hario & Rintala, 2009)	Individuals hatched in years with low habitat quality took longer to enter the breeding population (Hario & Rintala, 2009)		
Distributional measures				
Phenology	Length of breeding period (Raquel et al., 2016) Residence times on non‐breeding or stopover sites (O'Neal et al., 2012; Rice et al., 2007; Williams et al., 2019)	How late in a season a females pass through a postbreeding migration staging site is correlated with the production of juveniles in the previous breeding season (McKinnon et al., 2022)	Spring migration stopover duration can decrease as a function of Julian day of the year (Williams et al., 2019) Waterbirds may be able to regulate timing of some processes such as the completion of molt to ensure that they occur at the appropriate time in the annual cycle even when other components of the annual cycle are delayed (Marmillot et al., 2016)	Site/region—within season/annual
Age class distribution	Age class distribution (Fernández & Lank, 2010)	Adult shorebirds occupy sites with greater prey availability and lower predation risk than immature birds (Fernández & Lank, 2006)	Contrary to theory, juveniles may occupy regions with higher survival probability and move to regions where survival probability is lower as they reach adulthood (Lok et al., 2011)	Site/region—instantaneous/within season
Hunting records	Harvest numbers as an indicator of present and past habitat quality (Merendino et al., 1992)	The number of ducks harvested correlates with variables relating to wetland availability (Moloney et al., 2022)	—	Region—annual
Individual condition measures				
Morphological variables	Abdominal profile index (Swift et al., 2020) Body mass (Herring & Gawlik, 2013; Hunt et al., 2017) Body condition index (Aubry et al., 2013; Parks et al., 2016) Chick growth rate (Hunt et al., 2017; Owen & Pierce, 2014)	Abdominal profile index on the nonbreeding grounds was correlated with breeding ground return rates, and subsequent nest survival and chick fate (Swift et al., 2020) Chick growth rates and adult body mass were positively correlated with invertebrate abundance in breeding Piping Plovers (Hunt et al., 2017) Body condition is positively correlated with survival (Fleskes et al., 2002)	Abdominal profile index can be correlated with reproductive performance in productive years, but in unproductive years stochastic effects (e.g., nest predation) can confound the relationship (Klaassen et al., 2017) Body condition may be independent of food availability if foraging effort can be increased to buffer declines in food availability (Cope, 2003) Individuals may increase fat stores in landscapes where food resources are unpredictable to buffer against periods of food shortage (Ruthrauff et al., 2013)	Site/region—within season/annual
Physiological variables	Stress markers*** (Aharon‐Rotman, Buchanan, et al., 2016; Herring & Gawlik, 2013; Thomas & Swanson, 2013) Immune response markers (Buehler et al., 2009) Foraging metabolites (Lyons et al., 2008; Thomas & Swanson, 2013)	Birds that occupy sites with higher fuelling rates have lower concentration of physiological markers of stress in their blood (Aharon‐Rotman, Buchanan, et al., 2016)	Different species with different foraging strategies can have different blood physiology responses to changing availability of prey (Herring & Gawlik, 2013) Hemoglobin concentration does not correlate with breeding territory settlement decisions in terms of a territory's expected reproductive output (Minias & Janiszewski, 2016)	Site/region –Within season/annual
Parasite burden	Intestinal helminth load (Conner England et al., 2018) Haemosporidian parasite infection (Aharon‐Rotman, Buchanan, et al., 2016)	Haemosporidian blood parasite infection had a negative effect on body fat levels (Merrill et al., 2018)	Parasite burden negatively correlated with foraging habitat quality for some parasite taxa, but not significantly for all parasite taxa (Conner England et al., 2018) Parasite occurrence was not correlated with body condition, and the long lifespan of some parasites can mean that patterns of parasite burden reflect habitat use prior to arrival in the study area (Haukos & Neaville, 2003)	Site/region –Within season/annual
Ptilochronology	Feather growth rate (Swift et al., 2020)	Width of feather growth bands was positively correlated with an index of body condition (abdominal profile index) and feeding rates (Swift et al., 2020)	—	Site/region –Within season/annual
Behavioral measures				
Foraging parameters	Peck/probe rate (Castillo‐Guerrero et al., 2009; Mander et al., 2013) Success rate (Castillo‐Guerrero et al., 2009; Swift et al., 2020) Step rate during foraging (Mander et al., 2013) Energy intake rate (Yu et al., 2020)	Positively correlated with prey density and biomass, and at productive sites may not be affected by interference competition (Lourenço et al., 2005; Rose & Nol, 2010) Peck rate is correlated with defecation rate indicating that peck rate is a meaningful proxy for intake rate (Rose & Nol, 2010) Where foraging success can be quantified, the foraging parameters peck rate, step rate, and turning rate all conform with theoretical expectations (positive correlation for peck rate and turning rate, negative correlation for step rate) (Lourenço et al., 2005)	Capture success can be influenced by conspecifics, with increases in capture success occurring until conspecific density becomes high enough to induce interference competition (Stolen et al., 2012) Foraging rate (e.g., peck rate or submergence duration) have an asymptotic relationship with food availability, so may not be a true indication of habitat quality in very high‐productivity landscapes (Nolet et al., 2007; Rose & Nol, 2010) Pecking rate can be significantly higher than probing rate for an equivalent energy return (Kuwae et al., 2010) Peck rate increases as habitat quality declines for geese (Pezzanite et al., 2005) In tactile‐foraging species, peck rate may be a poor indicator of habitat quality because success rate is governed by prey density rather than number of pecks (Guerra et al., 2016)	Site/region – Instantaneous/within season/annual
Time budgets	Proportion of time spent foraging (Castillo‐Guerrero et al., 2009; Dugger & Feddersen, 2009; van der Kolk et al., 2019) Proportion of time in non‐foraging behaviors (e.g., vigilance, disturbance) (Castillo‐Guerrero et al., 2009; Maslo et al., 2012; Yu et al., 2020)	Oystercatchers that spent longer foraging had lower inferred survival (van der Kolk et al., 2019) Human disturbance decreases time spent foraging and increases time allocated to escape behaviors (Cheng et al., 2018)	Time budgets may vary within an individual period of the annual cycle (e.g., between breeding stages, or within the nonbreeding period) (Castillo‐Guerrero et al., 2009; Mallory et al., 1999) or due to the presence of conspecifics (Kosztolányi et al., 2006; Mallory et al., 1999) Time budgets may vary among individuals in response to the same environmental conditions owing to individual specializations(van der Kolk et al., 2019)	Site/region – Instantaneous/within season/annual
Anti‐predator behaviors	Vigilance rates (Fernández & Lank, 2010) Flight initiation distance (Gunness et al., 2001)	At sites where vigilance rates were higher, waterbirds maintained lower body mass (Fernández & Lank, 2010)	Flight initiation distances can change without changes in predation pressure owing to seasonal variation in the density of heterospecific birds that produce alarm calls (Koivula & Rönkä, 1998)	Site/region – Instantaneous/within season/annual
Individual movements	Home range size (Herring & Collazo, 2005) Commuting distance (Custer et al., 2004) Site fidelity (Jackson, 1994)	Home range size is negatively correlated with reproductive output (Reynolds et al., 2012) Birds from breeding sites that had poor reproductive output were more likely to disperse, dispersed over greater distances, and were more likely to settle in sites that had higher reproductive output the year before (Jackson, 1994)	Home range size may not be related to food availability (Jourdan et al., 2021) Home range size can differ between sexes (Schlägel & Mädlow, 2022) Breeding site fidelity may not be a useful indicator at the individual level due to stochastic events (requires population‐level data) (Blums et al., 2002) Breeding site fidelity is likely to be a poor indicator of habitat quality in species with high breeding success due to insufficient variation across the population (Blums et al., 2002) No effect of nesting success in one year and the distance to the nest site the following year (Lecomte et al., 2008) Site fidelity on wintering grounds increased with age, but adults settled progressively into sites with low survival (Lok et al., 2011)	Site/region – Instantaneous/within season/annual
Flight speeds	Flight speeds between foraging patches (Reurink et al., 2015)	Birds fly faster when heading to patches of high prey abundance because the greater expected returns are able to offset the greater flight costs of choosing to fly faster (Reurink et al., 2015)	Requires the birds to have perfect knowledge of the resource distribution available (Reurink et al., 2015), which may not always be the case (Lewis et al., 2010)	Site/region – Instantaneous/within season/annual
Parental investment	Nest attendance patterns Clutch mass (Hunt et al., 2017) Brood abandonment (Bustnes et al., 2002)	Clutch mass was positively associated with prey availability (Hunt et al., 2017) Females in good body condition were less likely to abandon their brood (Bustnes et al., 2002) Incubation stints were shorter at sites where human disturbance levels were high (Cheng et al., 2018)	Contrary to theory, parental investment may increase in favorable conditions because density‐dependent effects require parents to protect the brood from conspecifics for longer (Kosztolányi et al., 2006) Contrary to theory, females invested less in brood defense when conditions were good, likely because females gain greater survival benefit from the good conditions than their offspring and hence behave to maximize future reproduction (Gunness et al., 2001) The influence of nest attendance patterns on reproductive output can vary dependent on the spatial configuration of the habitat and the way that that influences foraging time (Thibault et al., 2010) The effect of environmental variability on patterns in nest attendance can be dependent on reproductive strategy (biparental care vs. uniparental care) (Meyer et al., 2021)	

*Note*: A ‘—’ symbol in the supporting evidence and contradictory evidence columns indicates that no data for these cells were found in the reviewed papers. The spatial (site, region, and flyway) and temporal (instantaneous, within‐season, annual) scales that data collection pertains to are also given.

In many cases, the proxies used assumed a simplistic relationship between the measured attribute and habitat quality that ignored other potential influences on true habitat quality. For example, studies that measured prey intake rate assumed that higher prey intake rates equated to better quality habitat without explicitly considering factors such as predation risk or the physiological costs of inhabiting sites with profitable foraging conditions. This is an important consideration that practitioners must evaluate when determining which variables they will measure and the reliability of their findings.

**FIGURE 1:**
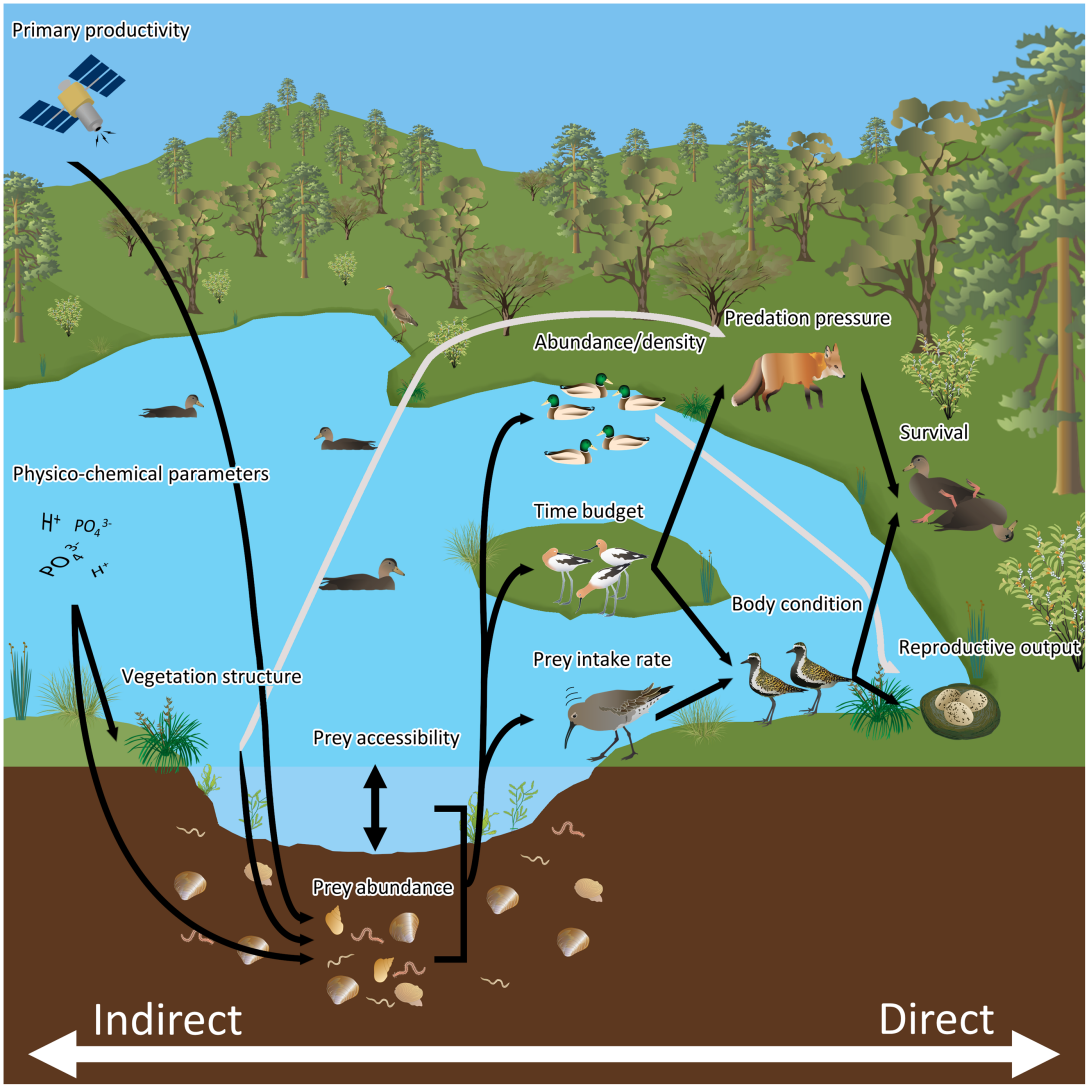
An example of the hierarchy among a selection of proxy methods for assessing waterbird habitat quality. The horizontal axis shows differences in terms of how directly or indirectly a selected habitat quality proxy relates to the ultimate determinants of habitat quality: reproductive output and survival probability. Arrows point in the direction of the presumed relationship from causal factor to outcome. Arrows originating from a line linking two proxy measures together indicate that the outcome results from the interaction between the two linked factors. Gray arrows depict relationships where the link likely manifests itself via another pathway in the ecological web. For example, greater waterbird abundance/density does not necessarily increase the reproductive output of an individual. Rather, more individuals are probably drawn to sites with high prey abundance and accessibility, where prey intake rates and thus waterbird body condition and then reproductive output can be maximized. Artworks for some proxy measures were obtained from the Integration and Application Network Media Library (ian.umces.edu/media‐library), with contributions from artists Tracey Saxby, Kim Kraeer, Lucy Van Essen‐Fishman, Dieter Tracey (Marine Botany UQ), Joanna Woerner, Emily Nastase, Jason C. Fisher (University of California Los Angeles), Jane Hawkey, and Jane Thomas.

The reviewed studies often presented evidence for a hierarchy in terms of how relevant each proxy was for assessing habitat quality. Some proxies were used more often as response variables whereas others were used consistently as predictor variables. Proxies often used as response variables (e.g., measures of reproductive output, survival, abundance, body condition) were typically more directly linked to demographic parameters, whereas proxies relating to physico‐chemical properties of wetlands (e.g., vegetation structure, primary productivity, nutrient concentrations) seldom featured as response variables. We interpret this as an indication that managers seeking to assess habitat quality are best equipped if they are able to measure proxies most directly related to demographic parameters (Figure 1).

### 3.1 Methods of habitat quality assessment

#### 3.1.1 Measuring demographic parameters

By definition, the quantification of habitat quality depends on estimating a site's contribution to survival and reproduction. Therefore, any method that directly measures survival or reproductive output will produce a quantitative estimate of the respective parameter that is free from error propagation caused by imperfect correlation between a measured attribute and these variables. Consequently, studies that directly measure both of these parameters are likely to provide the most accurate quantification of habitat quality with the fewest assumptions about the direction and strength of correlations between the measured attribute and population growth rate. However, cautious interpretation is still required when only one of these attributes is measured, because sites with similar reproductive output can have divergent population trajectories if the population size is governed by adult survival, and vice versa (Cohen et al., 2009). Similarly, emigration or immigration at a site may also obscure the signal arising from measures of reproduction and survival (Cohen et al., 2009). Caution must also be used if the measure of reproductive output reflects an early stage in the reproductive cycle (e.g., number of breeding pairs, clutch size) because the number of individuals that recruit into the breeding population from these nesting attempts may not be perfectly correlated with this early season data. For example, density‐dependent effects may mean that the number of pairs increases but the number of chicks fledged per pair declines (Kwon et al., 2021). Measuring demographic rates can be a lengthy, costly, and logistically challenging process. In waterbird research, directly measuring a site's contribution to survival and reproduction may be unachievable owing to the mobility of waterbird populations. Although our structured search returned examples of studies that did quantify survival (e.g., Alves et al., 2013; Rice et al., 2007; Swift et al., 2020) and/or reproduction (e.g., Hunt et al., 2017; Powell & Powell, 1986; Swift et al., 2020), most studies used proxies for one or both of these measures.

#### 3.1.2 Estimating food abundance and availability

Many of the proxies in the reviewed studies assumed that a high‐quality habitat provided waterbird individuals with a high net energy intake rate. The corollary assumption was that high net energy intake results in increased survival and reproductive performance. Methods used to infer net energy intake rate included measures of prey abundance, prey accessibility, and waterbird physiology or morphology as an indicator of past foraging returns (Table 1). Habitat quality assessments that are based on habitat attributes are appealing because results are independent of variation in bird behavior caused by factors unrelated to local habitat quality (e.g., current wind and rain conditions can determine which sites waterbirds use at very local scales (Kelly, 2001) and these short term changes are not typically useful for managers). For this reason, measuring the abundance or biomass of food was used widely in the reviewed studies to assess waterbird habitat quality. There is support for this method being an appropriate proxy for habitat quality because waterbirds preferentially forage at sites with the highest prey biomass and density (Guerra et al., 2016; Rose & Nol, 2010). Moreover, prey availability has a positive influence on reproductive performance and survival (Herring et al., 2010; Holopainen et al., 2014; Swift et al., 2020). However, there are also situations where prey biomass at a site can be a poor indicator of habitat quality. For example, sites with high prey biomass are not always favored foraging sites (Hagy & Kaminski, 2015), and although these sites might have high occupancy, they do not necessarily support high waterbird abundance (Gillespie & Fontaine, 2017). This suggests that factors such as predation risk, forager condition, and prey accessibility modulate the effect of prey biomass on habitat quality (Hagy & Kaminski, 2015). Such a relationship is also dependent on waterbirds having a perfect knowledge of the distribution of prey resources (Reurink et al., 2015), which may not always be the case (Lewis et al., 2010), and relies on researchers correctly identifying dietary preferences and requirements of focal species.

The presence of suitable water levels and variation in water levels was also used as a proxy for habitat quality in the reviewed studies. These habitat attributes can influence accessibility of prey and foraging energetics (Herring & Gawlik, 2013; Kang & King, 2014). In some cases, only a small proportion of a wetland provides suitable water levels for waterbirds to access prey (Collazo et al., 2002). This suggests that there is value in quantifying either prey biomass or the amount of suitable habitat through water level measurements. However, the two attributes will interact to influence the net rate of energy intake possible at a site meaning studies that measure both variables may have a greater likelihood of teasing apart meaningful habitat quality relationships and informing appropriate management (Herring & Gawlik, 2013). Similarly, remotely sensed measures of primary productivity (e.g., Normalized Difference Vegetation Index) are expected to be correlated with prey abundance. Yet, the relationship between net energy intake rate and primary productivity is dependent on changes in primary productivity causing changes in prey abundance (e.g., invertebrates, seeds, tubers) as well as those prey items being available to feeding waterbirds (Guan et al., 2016; Zhang et al., 2017). This suggests there is a hierarchy in the ability of proxies to provide precise habitat quality estimates based on how direct the link between the variable being measured and net energy intake rates is (Figure 1). In addition to this hierarchy, the precision of the proxy used may also be dependent on other factors, such as predation risk, that are not encompassed within the scope of the proxy measure but do influence true habitat quality.

#### 3.1.3 Estimating food intake rate

The behavior and habitat use patterns of waterbirds themselves were often used in the reviewed studies to infer underlying patterns of habitat quality (Table 1). Indicators of prey intake rate (be it current, past or expected future foraging returns) were frequently used metrics of habitat quality. Variables including peck rate, capture success rate, and the proportion of time a bird spent foraging were commonly measured to assess the current rate of energy intake supported by a habitat. Defecation rate is significantly correlated with peck rate in a visually foraging shorebird, supporting the assumption that peck rate represents a valid indicator of intake rate (Rose & Nol, 2010). Likewise, sites with a higher peck rate or probe rate had a higher rate of successful prey captures in a study where capture success could be visually verified (Kuwae et al., 2010). However, different prey items have different energy content and different processing costs within the digestive system (Dugger et al., 2007; Jorde et al., 1995). This means that the net rate of energy intake will depend on the prey type consumed. This may not be an issue in studies of diet specialists, but it may confound the interpretation of peck rate and capture success data for diet generalists. In situations where the diet of the population being studied is not well understood, investigating the prey community composition to determine prey encounter rates, or dietary studies (e.g., metabarcoding of prey DNA sequences in fecal samples) will inform whether differences in peck rate between sites or across time genuinely reflect changes in energy returns.

Intake rates over the recent and more distant past were inferred from a variety of variables including body condition, blood metabolites, and indicators of feather growth rate. These have the advantage that they reflect assimilated energy rather than gross intake including energy lost via excretion or through processing costs. However, the longer timeframe of integration meant that studies using these methods were rarely site‐specific, rather they tended to assess habitat quality at regional scales (e.g., Aharon‐Rotman, Buchanan, et al., 2016). In cases where individuals use only a small geographic area (e.g., when nesting constrains movements, or individuals have strong residency patterns) these measures may provide insights into site‐specific habitat quality. For example, Swift et al. (2020) found that visually scored body condition of nonbreeding Hudsonian Godwits *Limosa haemastica* was correlated with pecking rate at individual nonbreeding sites. This suggests that these birds were resident at sites long enough to integrate site‐specific habitat quality information in the form of body condition. Importantly, birds with higher body condition had higher survival and reproductive output the following breeding season, indicating that body condition reliably influenced demographic rates (Swift et al., 2020). Conversely, body condition may not be a sensitive enough parameter in some cases to truly reflect underlying changes in habitat quality. For example, Gunness et al. (2001) detected no seasonal change in duckling survival for mothers with high body condition, but broods of mothers with low body condition did have reduced survival late in the season. This suggests that there is some buffering capacity for females with high body condition to moderate the effects of seasonal declines in habitat quality. Furthermore, individuals inhabiting sites with variable prey availability may store more energy reserves than individuals with consistent access to prey because they must maintain enough energy reserves to see them through periods of resource scarcity (Ruthrauff et al., 2013). For this reason, it may be challenging to infer patterns of habitat quality from body condition alone.

#### 3.1.4 Predation pressure

Given the direct link between predation pressure and survival, it was surprising that predation pressure was estimated relatively infrequently in the reviewed studies. This is perhaps reflective of the difficulties of censusing predator populations due to predators of waterbirds typically occurring at low density and predation events on adult waterbirds being rare. Where predation pressure was quantified, these studies often focused on nest predation (e.g., Kenow et al., 2009; Pehlak & Lõhmus, 2008; Trinder et al., 2009). Most studies that inferred an influence of predation pressure on habitat quality assumed that the abundance of predators was correlated with predation rate without explicitly testing this assumption, which may be problematic when generalist predators are involved. Some studies also assessed predation pressure by using vigilance or escape behaviors of waterbirds (Fernández & Lank, 2010; Gunness et al., 2001). This has the advantage of integrating information on the degree of lost foraging time as a result of predation pressure because lost foraging opportunities will affect reproductive performance as well as survival (Castillo‐Guerrero et al., 2009).

#### 3.1.5 Physical habitat attributes

Many of the reviewed studies measured various physical and/or chemical attributes of waterbird habitats to infer habitat quality. The attributes measured were purported to influence habitat quality via their contribution to supporting viable prey populations (e.g., water pH, water conductivity, sediment grain size), enabling access to sufficient quantities of food (e.g., water area, pond density in the local area and vegetation composition, as well as water level which we discussed previously), or providing shelter from predators (e.g., vegetation structure). In most cases, these environment attributes are linked indirectly to demographic rates (Figure 1) and the mechanisms governing their effects may be difficult to disentangle (Raquel et al., 2016). Nonetheless, physical attributes of the habitat may provide waterbirds with visual cues as to the quality of a site and play a role in determining patterns of site use, which can have flow‐on effects on demographic rates (Buderman et al., 2020).

#### 3.1.6 Species richness

Among the reviewed studies, some used species richness as an indicator of habitat quality (e.g., Arzel et al., 2015; Dugger & Feddersen, 2009; Hickman, 1994). As established above (Section 1), habitat quality is a species‐specific parameter. Hence, species richness does not necessarily align with the definition of habitat quality unless it is viewed as an emergent property of a system that is able to provide high‐quality habitat for an array of species. There are some circumstances whereby the presence of heterospecific individuals may enhance habitat quality for certain species (Arzel et al., 2015). For example, the abundance of gulls (*Larus* spp.) was correlated with better waterbird brood success, probably because gulls provided defense against predators (Arzel et al., 2015; but see Broyer (2009) where no such effect of gulls was found). However, facilitative interactions will not occur in all species pairings and some interactions will have a negative impact on habitat quality for one species, such as aggression by Mute Swans (*Cygnus olor*) leading to a reduction in breeding pair density of Mallards (*Anas platyrhynchos*; Broyer, 2009).

#### 3.1.7 Climate, weather, and terrain attributes

A feature of many of the reviewed studies was the inclusion of climate, weather, and terrain attributes. These variables generally cannot be influenced by wetland managers, so on face value they may seem less important for the study of habitat quality from a management perspective. Nonetheless, they can play a key role in wetland availability (e.g., rainfall) and can also directly influence energy budgets (e.g., temperature and wind effects on thermoregulation), which have important ramifications for whether a site or region represents suitable habitat (Krijgsveld et al., 2003; McKinney & McWilliams, 2005; Sorenson et al., 1998). Furthermore, these parameters can provide contextual information that helps explain some of the variation in relationships between other parameters of management interest. For example, survival and fecundity of Eurasian Oystercatchers (*Haematopus ostralegus*) with a bill morphology suited to generalist foraging is high in most years, but in years with a harsh winter their survival is reduced relative to individuals with a bill morphology suited to specialized foraging strategies (van de Pol, Brouwer, et al., 2010). The addition of climate information to van de Pol, Brouwer, et al. (2010) study provides insight into why measurements of short‐term prey intake rates do not align with long‐term fitness outcomes for individuals. As a consequence of the influence of climate, weather, and terrain variables on habitat quality, they may provide information relevant to management prioritization decisions. For example, species that are restricted to a narrow elevation range may be more sensitive to changes in habitat quality and may benefit most from management to improve habitat conditions (Xu et al., 2019). Given the ready availability of datasets relating to climate, weather, and terrain in most cases (e.g., global climate models, local weather stations, and high resolution digital elevation models) the ability to include these variables as covariates provide researchers with additional insights to disentangle the complex interactions influencing waterbird habitat quality.

#### 3.1.8 Other methods

A variety of other methods were used infrequently in the reviewed studies (Table 1). These included estimates of levels of human disturbance, individual movement data (e.g., home range size), and the spatial distribution of individuals in different age classes. Despite their infrequent use, these methods may provide meaningful habitat quality information. Factors such as the cost of obtaining the data or the difficulty of obtaining the data (e.g., challenges distinguishing between age classes in the field) probably contributed to their infrequent use.

#### 3.1.9 Combination of methods

Many of the reviewed studies recorded data on multiple proxies for habitat quality. Multiple lines of evidence allowed researchers to tease apart complex relationships among various parameters in their respective study systems and provide powerful insight to conservation managers (Cohen et al., 2009; Hunt et al., 2017; Swift et al., 2020). In these studies, it was often possible to pinpoint factors that were limiting habitat quality, providing managers with priorities to address in order to improve habitat quality. For example, Cohen et al. (2009) recommended that restoring Piping Plover, *Charadrius melodus*, habitat adjacent to bayside intertidal flats would improve habitat quality by increasing the number of breeding pairs that could occupy a site. However, this action must be carried out in conjunction with predator management in order to achieve the desired increase in reproductive output.

##### Applications

A feature of many of the reviewed studies was the use of chosen habitat quality proxy variable(s) as inputs into sophisticated ecological analyses, such as habitat suitability models (e.g., Guan et al., 2016; Hsu et al., 2014; Tang et al., 2016; Wen et al., 2016), carrying capacity estimates (e.g., Collazo et al., 2002; Mu et al., 2022; Stillman et al., 2000), population viability analyses (e.g., Mattsson et al., 2012; Saunders et al., 2021; Weegman et al., 2022), or to predict the outcome of management interventions on waterbird vital rates (e.g., Beerens et al., 2015; Toral et al., 2012). Where the predictor variables for these analytical approaches covered large spatial extents or could be sourced from extensive temporal archives, knowledge gained from site‐level studies could be extrapolated in space and/or time to provide a far‐reaching evaluation of habitat quality. Accordingly, many of the studies that produced a habitat suitability model used remotely sensed data sources. For example, Mokany et al. (2022) used satellite‐derived water cover data to predict habitat quality for waterbirds during two time periods across a study area spanning 1.06 million km^2^, and Wen et al. (2016) used waterbird presence and Normalized Difference Vegetation Index data to predict changes in waterbird distribution in response to drought‐breaking rains across a 810,000 km^2^ study area.

### 3.2 Factors influencing the choice of variables to measure

#### 3.2.1 Staying within the project's scope

Our synthesis of the habitat quality literature indicates that there is a hierarchy of data quality from directly monitoring demographic rates to measuring parameters that are increasingly indirectly linked to demography. Yet, practitioners typically face a trade‐off between the need for accuracy of the habitat quality estimate and their particular study's aims and constraints. If it is feasible, measuring demographic rates directly generally involves extended field time, individually marked birds, limited spatial scale, and substantial costs (Buderman et al., 2020). Other factors may also influence the suitability of a proxy for the habitat quality assessment at hand including ethical considerations (Hunt et al., 2013), and the availability of appropriately trained personnel. Physiological and morphological measurements used in the reviewed studies typically required birds to be handled (but see the abdominal profile index method; Swift et al., 2020), which imposes stress on the study subjects (Karlíková et al., 2018), and capturing a large sample size of birds can be time consuming. This may mean that methods requiring birds to be handled, including individually marking birds for quantifying demographic rates, are not feasible within the scope of a project.

#### Spatial and temporal scales of assessments

Another consideration that must be made prior to implementing a study on habitat quality is whether the habitat quality measure being used returns data at a relevant spatial and/or temporal scale. For example, prey abundance measures typically provide very local scale (both spatial and temporal) information on habitat conditions, but may not be representative of habitat quality across the entire wetland or extended timeframes (e.g., the entire nonbreeding period). For example, Fonseca and Navedo (2020) reported a 43% reduction in invertebrate prey biomass as a result of shorebird foraging in study plots over the course of 3 days. Consequently, habitat quality assessments either side of this three‐day period could yield vastly different inferences about local habitat quality and neither may be representative of habitat quality over an extended timeframe. The accuracy of these methods in terms of returning habitat quality data at timescales meaningful for management will therefore be increased by repeated sampling (Murray et al., 2010). This was reflected in a number of the reviewed studies, especially those aimed at specifying management regimes, repeating sampling both spatially, and intra‐ and inter‐annually (e.g., Gillespie & Fontaine, 2017). Whereas methods that relied on measuring attributes of the habitat typically provided snapshot estimates of habitat quality, methods reliant on waterbird body condition or physiology (e.g., abdominal profile index or red blood cell heat shock protein concentrations) often provide information integrated over longer timeframes (Herring & Gawlik, 2013). They may therefore be unsuitable for site‐specific and/or instantaneous habitat quality questions, but may be applied to questions informing management of a regional wetland complex over broader timeframes. Similarly, remotely sensed measures of primary productivity offer the potential to rapidly and cost‐effectively monitor habitat conditions at large spatial and temporal scales. For example, Wen et al. (2016) used remotely sensed primary productivity data to inform an assessment of waterbird habitat quality across a 810,000 km^2^ study area in multiple years.

There is no rule that governs whether the spatial or temporal scale of a particular proxy is appropriate for a particular application because even labour intensive or costly methods that return site‐specific information may be suitable for large‐scale projects if the budget enables sufficiently widespread sampling (e.g., sites and time points). We provide some recommendations as to the spatial and temporal scales that methods for habitat quality assessments are typically carried out at (Table 1). Readers may also find papers such as Behney et al. (2014) guide to determining the optimum number of benthic core samples to collect useful for planning how much field effort is likely to be involved when planning a sampling regime.

### 3.3 What makes for a good habitat quality assessment?

Measuring habitat quality enables conservation managers to assess the need for or effectiveness of management actions (e.g., Schultz et al., 2020). The ultimate objective of conservation management is to influence demographic parameters of conservation targets to improve conservation status. Therefore, assessments of habitat quality inherently must determine a site's contribution to survival probability and/or reproductive output. This requires there to be a link between the variable, or combination of variables, used to measure habitat quality and demographic rates (Figure 1). Before commencing an assessment of habitat quality, the researcher must carefully consider whether the selected measure does actually influence demographic rates. For example, quantifying the time budgets of waterbirds is a commonly used method for inferring differences in habitat quality (Dugger & Feddersen, 2009; van der Kolk et al., 2019). However, the inferences derived from time budget comparisons may not actually reflect changes in underlying habitat quality. Time budgets can be flexible to buffer intrinsic changes in requirements (Mallory et al., 1999). For example, this may be due to individuals dedicating more time to foraging to meet the metabolic demands of producing a clutch of eggs (Mallory et al., 1999), or dedicating more time to feeding to fatten up for migration (Castillo‐Guerrero et al., 2009). That is not to say that time budgets are unsuitable for quantifying habitat quality, but care must be taken to ensure that appropriate comparison groups are being used (e.g., sampling at the same time of year; Pezzanite et al., 2005). Similarly, care must be taken to ensure that the appropriate response variable is measured. For example, management may result in better nesting habitat, but no advantage may be conferred on subsequent chick survival (Smart et al., 2013). In this case, management may result in more chicks recruiting to the breeding population because there are more nests, but if managers were measuring the probability of a given chick fledging there would be no signal to indicate the improvement in population growth.

Researchers must also be aware that inferences made about populations that are not at equilibrium may depart from theoretical relationships underpinning many habitat quality proxies. For example, populations that have been reduced below carrying capacity by historical or offsite factors may not show any temporal differences in various local habitat quality proxies (e.g., foraging success, stress markers, body condition, time budgets) because individuals are easily able to meet their resource requirements even if local habitat quality is declining. Similarly, there may be differences in the relevance of some habitat quality proxies depending on whether the conservation target is a resident population, or a dispersive or migratory population (Loewenthal et al., 2015). Abundance and density are clearly linked to local habitat quality for resident populations, but may not be truly reflective of local habitat quality for populations that undertake large‐scale movements exposing individuals to factors that limit population size elsewhere in the range. For example, Jia et al. (2018) reported declines in abundance of migratory shorebirds at a migratory staging site, but none of the measured proxy variables for habitat quality could explain these declines. They suggest that factors in other parts of the migratory range may be responsible for driving the observed declines in abundance rather than changes in habitat quality at their study site.

Many of the habitat quality proxies identified in this review assume individuals have perfect knowledge of the resource distribution at a site and behave such that the net rate of energy gain is being maximized at any given time (Reurink et al., 2015). Several factors can result in waterbirds using their habitat in ways that do not conform to these assumptions (Stillman et al., 2005). The choice of foraging site for many waterbirds is strongly influenced by conspecific attraction (Gawlik & Crozier, 2007; Herring et al., 2015; Smith, 1995). This is also true for the selection of nest sites (Sebastián‐González, Sánchez‐Zapata, Botella, & Otso, 2010). Furthermore, fidelity to areas that have provided favorable habitat conditions in the past may decouple patterns of waterbird habitat use from current habitat conditions (O'Neil et al., 2014). Waterbird habitat requirements may also change with breeding stage (Holopainen et al., 2014), and during less energetically demanding parts of the annual cycle, such as the nonbreeding period, individuals may be less selective in their habitat use decisions (Sebastián‐González, Sánchez‐Zapata, & Botella, 2010).

Most of the reviewed studies provided a relative assessment of habitat quality (i.e., they compared waterbird habitat quality at a site to previous points in time, or made comparisons between sites). These studies allow researchers to determine habitat quality trends or identify the best and worst sites in a landscape, but do not enable managers to determine whether the habitat quality is sufficient to maintain viable waterbird populations. There were some studies that sought to determine whether the habitat quality at a site was sufficient to support population growth or whether the site represented a sink habitat (e.g., Roy et al., 2019; Sabatier et al., 2010). These studies do enable managers to determine whether management intervention is necessary rather than arbitrarily setting a reference site as the standard against which to decide whether management is warranted. In particular, studies seeking to identify whether a site had sufficient habitat quality to support population growth tended to focus directly on reproductive output or survival data (Roy et al., 2019; Weiser et al., 2018a), or in some cases focused on energetic demands relative to prey resources (West et al., 2005).

Together, the potentially confounding factors mean that there is no universally applicable habitat quality proxy. Yet, with careful consideration and a detailed understanding of the ecology of the study system, waterbird researchers and management practitioners can derive meaningful measures of habitat quality.

### 3.4 Limitations of the review

We used only English language search terms. This could have resulted in some methods that are used in only a subset of countries that are non‐English‐speaking being overlooked by our research. Overlooking some methods should not bias the inferences we have drawn about methods that we did catalogue because evidence within the reviewed studies supporting or contradicting the use of a particular method remains valid. Furthermore, the mobility of many waterbirds and the frequency with which they cross international borders also promotes international collaboration along flyways as evidenced by groups such as the Atlantic Flyway Shorebird Initiative and the East Asian‐Australasian Flyway Partnership. These groups share knowledge among partners in countries flyway‐wide, so it is unlikely that a method that is useful in one part of the flyway would not be used throughout the flyway. Given that seven of the nine flyways recognized by Wetlands International (https://wpp.wetlands.org/background/WAF) include English‐speaking nations, we feel that this is unlikely to have resulted in us overlooking regionally specific methods to a large degree.

## 4 CONCLUSIONS

This review of the literature comprising more than 600 articles strongly suggests that there is no one broadly accepted method for assessing waterbird habitat quality. Directly measuring breeding success and survival rate are the most reliable measures, but it is unfeasible to obtain these data in many cases. A variety of proxy measures are available, but their interpretation requires substantive contextualization and a good understanding of their appropriateness to a specific project aim.

In general, if it is not possible to measure direct demographic parameters, projects should consider the suite of available proxy measures (Table 1) and consider which are most suitable to their site, budget and timeframe. Often, developing a protocol based on multiple proxies will increase confidence in results over the use of a single proxy. For example, studies investigating the comparative habitat quality of multiple sites could use a combination of waterbird abundance, behavior and body condition coupled with a measure of prey availability to gain insight into which site(s) are providing better food resources. Studies assessing if a single site is profitable for waterbirds from an energy perspective (i.e., habitat quality is sufficiently high to support population growth) could use a combination of waterbird behavior and available energy density to assess whether daily energy requirements are being met at the site. All studies using proxy measures should be mindful of the potential for interactions between features of the habitat (e.g., prey abundance and prey accessibility) to influence the direction of the relationship between habitat conditions and resultant demographic rates.

## AUTHOR CONTRIBUTIONS

**Rowan Mott:** Conceptualization (equal); data curation (equal); investigation (equal); methodology (equal); writing – original draft (lead); writing – review and editing (equal). **Thomas A. A. Prowse:** Conceptualization (equal); funding acquisition (equal); investigation (equal); methodology (equal); writing – original draft (equal); writing – review and editing (equal). **Micha V. Jackson:** Conceptualization (equal); investigation (equal); writing – original draft (equal); writing – review and editing (equal). **Daniel J. Rogers:** Funding acquisition (equal); writing – original draft (equal); writing – review and editing (equal). **Jody A. O'Connor:** Funding acquisition (equal); writing – original draft (equal); writing – review and editing (equal). **Justin D. Brookes:** Funding acquisition (equal); writing – original draft (equal); writing – review and editing (equal). **Phillip Cassey:** Funding acquisition (equal); writing – original draft (equal); writing – review and editing (equal).

## CONFLICT OF INTEREST STATEMENT

The authors have no conflict of interest to declare.

## Supporting information


Appendix S1

## Data Availability

Data are available in supplementary material.

## References

[ece39905-bib-0001] Aharon‐Rotman, Y. , Bauer, S. , & Klaassen, M. (2016). A chain is as strong as its weakest link: Assessing the consequences of habitat loss and degradation in a long‐distance migratory shorebird. Emu – Austral Ornithology, 116, 199–207.

[ece39905-bib-0002] Aharon‐Rotman, Y. , Buchanan, K. L. , Clark, N. J. , Klaassen, M. , & Buttemer, W. A. (2016). Why fly the extra mile? Using stress biomarkers to assess wintering habitat quality in migratory shorebirds. Oecologia, 182, 385–395. 27337963 10.1007/s00442-016-3679-1

[ece39905-bib-0003] Alves, J. A. , Gunnarsson, T. G. , Hayhow, D. B. , Appleton, G. F. , Potts, P. M. , Sutherland, W. J. , & Gill, J. A. (2013). Costs, benefits, and fitness consequences of different migratory strategies. Ecology, 94, 11–17. 23600235 10.1890/12-0737.1

[ece39905-bib-0004] Arzel, C. , Rönkä, M. , Tolvanen, H. , Aarras, N. , Kamppinen, M. , & Vihervaara, P. (2015). Species diversity, abundance and brood numbers of breeding waterbirds in relation to habitat properties in an agricultural watershed. Annales Zoologici Fennici, 52, 17–32.

[ece39905-bib-0005] Atiénzar, F. , Antón‐Pardo, M. , Armengol, X. , & Barba, E. (2012). Distribution of the white‐headed duck *Oxyura leucocephala* is affected by environmental factors in a Mediterranean wetland. Zoological Studies, 51, 783–792.

[ece39905-bib-0006] Aubry, L. M. , Rockwell, R. F. , Cooch, E. G. , Brook, R. W. , Mulder, C. P. H. , & Koons, D. N. (2013). Climate change, phenology, and habitat degradation: Drivers of gosling body condition and juvenile survival in lesser snow geese. Global Change Biology, 19, 149–160. 23504727 10.1111/gcb.12013

[ece39905-bib-0007] Austin, J. E. , Buhl, T. K. , Guntenspergen, G. R. , Norling, W. , & Sklebar, H. T. (2001). Duck populations as indicators of landscape condition in the prairie pothole region. Environmental Monitoring and Assessment, 69, 29–48. 11393543 10.1023/a:1010748527667

[ece39905-bib-0008] Austin, J. E. , O'Neil, S. T. , & Warren, J. M. (2017). Habitat selection by postbreeding female diving ducks: Influence of habitat attributes and conspecifics. Journal of Avian Biology, 48, 295–308.

[ece39905-bib-0009] Baker, J. D. , & Thompson, P. M. (2007). Temporal and spatial variation in age‐specific survival rates of a long‐lived mammal, the Hawaiian monk seal. Proceedings of the Royal Society B: Biological Sciences, 274, 407–415. 10.1098/rspb.2006.3737 PMC1702379 17164205

[ece39905-bib-0010] Barati, A. , Ataei, F. , Esfandabad, B. S. , Shabanian, N. , & Etezadifar, F. (2011). Variations in nest‐site parameters in breeding waterbirds at Lake Zarivar, western Iran. Avian Biology Research, 4, 87–92.

[ece39905-bib-0011] Barnett, C. A. , Suzuki, T. N. , Sakaluk, S. K. , & Thompson, C. F. (2015). Mass‐based condition measures and their relationship with fitness: in what condition is condition? Journal of zoology (London, England: 1987), 296, 1–5. 26019406 10.1111/jzo.12213 PMC4442632

[ece39905-bib-0012] Beerens, J. M. , Frederick, P. C. , Noonburg, E. G. , & Gawlik, D. E. (2015). Determining habitat quality for species that demonstrate dynamic habitat selection. Ecology and Evolution, 5, 5685–5697. 27069617 10.1002/ece3.1813 PMC4813099

[ece39905-bib-0013] Behney, A. C. , O'shaughnessy, R. , Eichholz, M. W. , & Stafford, J. D. (2014). Influence of item distribution pattern and abundance on efficiency of benthic core sampling. Wetlands, 34, 1109–1121.

[ece39905-bib-0014] Benedict, R. J. , & Hepp, G. R. (2000). Wintering waterbird use of two aquatic plant habitats in a southern reservoir. The Journal of Wildlife Management, 64, 269–278.

[ece39905-bib-0015] Blums, P. , Nichols, J. D. , Hines, J. E. , & Mednis, A. (2002). Sources of variation in survival and breeding site fidelity in three species of European ducks. Journal of Animal Ecology, 71, 438–450.

[ece39905-bib-0016] Boyer, R. A. , Coluccy, J. M. , Montgomery, R. A. , Redilla, K. M. , & Winterstein, S. R. (2018). The effect of habitat on the breeding season survival of mallards (*Anas platyrhynchos*) in the Great Lakes region. Canadian Journal of Zoology, 96, 700–706.

[ece39905-bib-0017] Broyer, J. (2009). Compared distribution within a disturbed fishpond ecosystem of breeding ducks and bird species indicators of habitat quality. Journal of Ornithology, 150, 761–768.

[ece39905-bib-0018] Bucher, E. H. , & Curto, E. (2012). Influence of long‐term climatic changes on breeding of the Chilean flamingo in mar Chiquita, Córdoba, Argentina. Hydrobiologia, 697, 127–137.

[ece39905-bib-0019] Buderman, F. E. , Devries, J. H. , & Koons, D. N. (2020). Changes in climate and land use interact to create an ecological trap in a migratory species. Journal of Animal Ecology, 89, 1961–1977. 32271949 10.1111/1365-2656.13228

[ece39905-bib-0020] Buehler, D. M. , Irene Tieleman, B. , & Piersma, T. (2009). Age and environment affect constitutive immune function in red knots (*Calidris canutus*). Journal of Ornithology, 150, 815–825.

[ece39905-bib-0021] Burton, N. H. K. , Armitage, M. J. S. , Musgrove, A. J. , & Rehfisch, M. M. (2002). Impacts of man‐made landscape features on numbers of estuarine waterbirds at low tide. Environmental Management, 30, 857–864. 12402099 10.1007/s00267-002-2732-5

[ece39905-bib-0022] Bustnes, J. O. , Erikstad, K. E. , & Bjorn, T. H. (2002). Body condition and brood abandonment in common eiders breeding in the high arctic. Waterbirds, 25, 63–66.

[ece39905-bib-0023] Castillo‐Guerrero, J. A. , Fernández, G. , Arellano, G. , & Mellink, E. (2009). Diurnal abundance, foraging behavior and habitat use by non‐breeding marbled godwits and willets at Guerrero Negro, Baja California Sur, México. Waterbirds, 32, 400–407.

[ece39905-bib-0024] Cheng, K. , Ayjan, Y. , Li, F. F. , & Zong, C. (2018). Behavioral responses of breeding common coots (*Fulica atra*) to recreational disturbancein the Anbang river nature reserve. Shengtai Xuebao, 38, 485–492.

[ece39905-bib-0025] Chyb, A. , & Minias, P. (2022). Complex associations of weather conditions with reproductive performance in urban population of a common waterbird. International Journal of Biometeorology, 66, 1163–1172. 35279734 10.1007/s00484-022-02266-6

[ece39905-bib-0026] Cleasby, I. R. , Bodey, T. W. , Vigfusdottir, F. , Mcdonald, J. L. , Mcelwaine, G. , Mackie, K. , Colhoun, K. , & Bearhop, S. (2017). Climatic conditions produce contrasting influences on demographic traits in a long‐distance Arctic migrant. Journal of Animal Ecology, 86, 285–295. 27973683 10.1111/1365-2656.12623

[ece39905-bib-0027] Cohen, J. B. , Houghton, L. M. , & Fraser, J. D. (2009). Nesting density and reproductive success of piping plovers in response to storm‐ and human‐created habitat changes. Wildlife Monographs, 173, 1–24.

[ece39905-bib-0028] Collazo, J. A. , Dawn, A. O. H. , & Kelly, C. A. (2002). Accessible habitat for shorebirds: Factors influencing its availability and conservation implications. Waterbirds, 25, 13–24.

[ece39905-bib-0029] Conner England, J. , Levengood, J. M. , Osborn, J. M. , Yetter, A. P. , Suski, C. D. , Cole, R. A. , & Hagy, H. M. (2018). Associations of intestinal helminth infections with health parameters of spring‐migrating female lesser scaup (*Aythya affinis*) in the upper Midwest, USA. Parasitology Research, 117, 1877–1890. 29696395 10.1007/s00436-018-5879-6

[ece39905-bib-0030] Cope, D. R. (2003). Variation in daily and seasonal foraging routines of non‐breeding barnacle geese (*Branta leucopsis*): Working harder does not overcome environmental constraints. Journal of Zoology, 260, 65–71.

[ece39905-bib-0031] Crowther, M. , Lim, W. , & Crowther, M. A. (2010). Systematic review and meta‐analysis methodology. Blood, 116, 3140–3146. 20656933 10.1182/blood-2010-05-280883

[ece39905-bib-0032] Cumming, G. S. , Paxton, M. , King, J. , & Beuster, H. (2012). Foraging guild membership explains variation in waterbird responses to the hydrological regime of an arid‐region flood‐pulse river in Namibia. Freshwater Biology, 57, 1202–1213.

[ece39905-bib-0033] Custer, C. M. , Suárez, S. A. , & Olsen, D. A. (2004). Feeding habitat characteristics of the great blue heron and great egret nesting along the upper Mississippi River, 1995‐1998. Waterbirds, 27, 454–468.

[ece39905-bib-0034] Davis, C. A. , & Smith, L. M. (1998). Ecology and management of migrant shorebirds in the Playa Lakes region of Texas. Wildlife Monographs, 140, 3–45.

[ece39905-bib-0035] Deboelpaep, E. , Coenegracht, T. , De Wolf, L. , Libert, A. , Vanschoenwinkel, B. , & Koedam, N. (2020). Bio‐energetic data show weak spatial but strong seasonal differences in wetland quality for waders in a Mediterranean migration bottleneck. Freshwater Biology, 65, 1529–1542.

[ece39905-bib-0036] Dickson, D. L. (1992). The red‐throated loon as an indicator of environmental quality . Occasional Paper – Canadian Wildlife Service, 73.

[ece39905-bib-0037] Dugger, B. D. , & Feddersen, J. C. (2009). Using river flow management to improve wetland habitat quality for waterfowl on the Mississippi River, USA. Wild, 59, 62–74.

[ece39905-bib-0038] Dugger, B. D. , Moore, M. L. , Finger, R. S. , & Petrie, M. J. (2007). True metabolizable energy for seeds of common moist‐soil plant species. Journal of Wildlife Management, 71, 1964–1967.

[ece39905-bib-0039] Duncan, P. , Hewison, A. J. M. , Houte, S. , Rosoux, R. , Tournebize, T. , Dubs, F. , Burel, F. , & Bretagnolle, V. (1999). Long‐term changes in agricultural practices and wildfowling in an internationally important wetland, and their effects on the guild of wintering ducks. Journal of Applied Ecology, 36, 11–23.

[ece39905-bib-0040] Féret, M. , Bêty, J. , Gauthier, G. , Giroux, J.‐F. , & Picard, G. (2005). Are abdominal profiles useful to assess body condition of spring staging greater snow geese? The Condor, 107, 694–702.

[ece39905-bib-0041] Fernández, G. , & Lank, D. B. (2006). Sex, age, and body size distributions of western sandpipers during the nonbreeding season with respect to local habitat. The Condor, 108, 547–557.

[ece39905-bib-0042] Fernández, G. , & Lank, D. B. (2010). Do sex and habitat differences in antipredator behavior of Western sandpipers *Calidris mauri* reflect cumulative or compensatory processes? Journal of Ornithology, 151, 665–672.

[ece39905-bib-0043] Fleskes, J. P. , Jarvis, R. L. , & Gilmer, D. S. (2002). September‐march survival of female northern pintails radiotagged in San Joaquin Valley, California. Journal of Wildlife Management, 66, 901–911.

[ece39905-bib-0044] Fletcher, K. , Aebischer, N. J. , Baines, D. , Foster, R. , & Hoodless, A. N. (2010). Changes in breeding success and abundance of ground‐nesting moorland birds in relation to the experimental deployment of legal predator control. Journal of Applied Ecology, 47, 263–272.

[ece39905-bib-0045] Fonseca, J. , & Navedo, J. G. (2020). Shorebird predation on benthic invertebrates after shrimp‐pond harvesting: Implications for semi‐intensive aquaculture management. Journal of Environmental Management, 262, 110290. 32090889 10.1016/j.jenvman.2020.110290

[ece39905-bib-0046] Ganzevles, W. , & Bredenbeek, J. (2005). Waders and waterbirds in the floodplains of the Logone, Cameroon and Chad, February 2000 . WIWO report 82. Beek‐Ubbergen, The Netherlands: Working Group of International Wader and Waterfowl Research (WIWO).

[ece39905-bib-0047] Gawlik, D. E. , & Crozier, G. E. (2007). A test of cues affecting habitat selection by wading birds. The Auk, 124, 1075–1082.

[ece39905-bib-0048] Ge, Z. M. , Wang, T. H. , Zhou, X. , Wang, K. Y. , & Shi, W. Y. (2007). Changes in the spatial distribution of migratory shorebirds along the Shanghai shoreline, China, between 1984 and 2004. Emu, 107, 19–27.

[ece39905-bib-0049] Gillespie, C. R. , & Fontaine, J. J. (2017). Shorebird stopover habitat decisions in a changing landscape. The Journal of Wildlife Management, 81, 1051–1062.

[ece39905-bib-0050] Godfrey, J. D. , Bryant, D. M. , & Williams, M. (2003). Energetics of blue ducks in rivers of differing physical and biological characteristics. Science for Conservation, 214, 35–68.

[ece39905-bib-0051] Guan, L. , Lei, J. , Zuo, A. , Zhang, H. , Lei, G. , & Wen, L. (2016). Optimizing the timing of water level recession for conservation of wintering geese in Dongting Lake, China. Ecological Engineering, 88, 90–98.

[ece39905-bib-0052] Guerra, A. S. , Micheli, F. , & Wood, C. L. (2016). Ecology of a vulnerable shorebird across a gradient of habitat alteration: Bristle‐thighed curlews (*Numenius tahitiensis*)(Aves: Charadriiformes) on Palmyra atoll. Pacific Science, 70, 159–174.

[ece39905-bib-0053] Guisan, A. , & Thuiller, W. (2005). Predicting species distribution: Offering more than simple habitat models. Ecology Letters, 8, 993–1009. 34517687 10.1111/j.1461-0248.2005.00792.x

[ece39905-bib-0054] Gunness, M. A. , Clark, R. G. , & Weatherhead, P. J. (2001). Counterintuitive parental investment by female dabbling ducks in response to variable habitat quality. Ecology, 82, 1151–1158.

[ece39905-bib-0055] Haddaway, N. R. , Bethel, A. , Dicks, L. V. , Koricheva, J. , Macura, B. , Petrokofsky, G. , Pullin, A. S. , Savilaakso, S. , & Stewart, G. B. (2020). Eight problems with literature reviews and how to fix them. Nature Ecology & Evolution, 4, 1582–1589. 33046871 10.1038/s41559-020-01295-x

[ece39905-bib-0056] Hagy, H. M. , & Kaminski, R. M. (2015). Determination of foraging thresholds and effects of application on energetic carrying capacity for waterfowl. PLoS One, 10, e0118349. 25790255 10.1371/journal.pone.0118349 PMC4366255

[ece39905-bib-0057] Hamza, F. , Hammouda, A. , & Selmi, S. (2015). Species richness patterns of waterbirds wintering in the Gulf of Gabès in relation to habitat and anthropogenic features. Estuarine, Coastal and Shelf Science, 165, 254–260.

[ece39905-bib-0058] Hario, M. , & Rintala, J. (2009). Age of first breeding in the common eider somateria m. mollissima population in the northern Baltic Sea. Ornis Fennica, 86, 81–88.

[ece39905-bib-0059] Haukos, D. A. , & Neaville, J. (2003). Spatial and temporal changes in prevalence of a cloacal cestode in wintering waterfowl along the Gulf coast of Texas. Journal of Wildlife Diseases, 39, 152–160. 12685079 10.7589/0090-3558-39.1.152

[ece39905-bib-0060] Hazlitt, S. L. (2001). Territory quality and reproductive success of black oystercatchers in British Columbia. Wilson Bulletin, 113, 404–409.

[ece39905-bib-0061] He, C.‐G. , Cui, L.‐J. , Ishikawa, T. , Sheng, L.‐X. , & Jiang, H.‐B. (2009). Study on the water regulation for preserving the nesting habitat of red‐crowned crane (*Grus japonensis* (Muller)). Journal of Northeast Normal University Natural Sciences Edition, 41, 105–111.

[ece39905-bib-0062] Herring, G. , & Collazo, J. A. (2005). Habitat use, movements and home range of wintering lesser Scaup in Florida. Waterbirds, 28, 71–78.

[ece39905-bib-0063] Herring, G. , & Gawlik, D. E. (2013). Differential physiological responses to prey availability by the great egret and white ibis. The Journal of Wildlife Management, 77, 58–67.

[ece39905-bib-0064] Herring, G. , Gawlik, D. E. , Cook, M. I. , & Beerens, J. M. (2010). Sensitivity of nesting great egrets (*Ardea alba*) and white ibises (*Eudocimus albus*) to reduced prey availability. The Auk, 127, 660–670.

[ece39905-bib-0065] Herring, G. , Herring, H. K. , & Gawlik, D. E. (2015). Social cues and environmental conditions influence foraging flight distances of breeding wood storks (*Mycteria americana*). Waterbirds, 38, 30–39.

[ece39905-bib-0066] Hickman, S. (1994). Improvement of habitat quality for nesting and migrating birds at the Des Plaines River wetlands demonstration project. Ecological Engineering, 3, 485–494.

[ece39905-bib-0067] Hierl, L. A. , Loftin, C. S. , Longcore, J. R. , Mcauley, D. G. , & Urban, D. L. (2007). A multivariate assessment of changes in wetland habitat for waterbirds at Moosehorn National Wildlife Refuge, Maine, USA. Wetlands, 27, 141–152.

[ece39905-bib-0068] Holopainen, S. , Nummi, P. , & Pöysä, H. (2014). Breeding in the stable boreal landscape: Lake habitat variability drives brood production in the teal (*Anas crecca*). Freshwater Biology, 59, 2621–2631.

[ece39905-bib-0069] Hsu, C.‐B. , Hwang, G.‐W. , Lu, J.‐F. , Chen, C.‐P. , Tao, H.‐H. , & Hsieh, H.‐L. (2014). Habitat characteristics of the wintering common teal in the Huajiang wetland, Taiwan. Wetlands, 34, 1207–1218.

[ece39905-bib-0070] Hu, C.‐S. , Song, X. , Ding, C.‐Q. , Ye, Y.‐X. , Qing, B.‐P. , & Wang, C. (2016). The size of winter‐flooded paddy fields no longer limits the foraging habitat use of the endangered crested ibis (*Nipponia nippon*) in winter. Zoological Science, 33, 345–351. 27498793 10.2108/zs150012

[ece39905-bib-0071] Hunt, K. L. , Catlin, D. H. , Felio, J. H. , & Fraser, J. D. (2013). Effect of capture frequency on the survival of piping plover chicks. Journal of Field Ornithology, 84, 299–303.

[ece39905-bib-0072] Hunt, K. L. , Fraser, J. D. , Karpanty, S. M. , & Catlin, D. H. (2017). Body condition of piping plovers (*Charadrius melodus*) and prey abundance on flood‐created habitat on the Missouri River, USA. The Wilson Journal of Ornithology, 129, 754–764.

[ece39905-bib-0073] Jackson, D. B. (1994). Breeding dispersal and site‐fidelity in three monogamous wader species in the Western isles, U.K. Ibis, 136, 463–473.

[ece39905-bib-0074] Jia, Q. , Wang, X. , Zhang, Y. , Cao, L. , & Fox, A. D. (2018). Drivers of waterbird communities and their declines on Yangtze River floodplain lakes. Biological Conservation, 218, 240–246.

[ece39905-bib-0075] Johnson, M. D. (2005). Habitat quality: a brief review for wildlife biologists. Transactions of the Western Section of the Wildlife Society, 41, 31–41.

[ece39905-bib-0076] Johnson, M. D. (2007). Measuring habitat quality: A review. The Condor, 109, 489–504.

[ece39905-bib-0077] Jorde, D. G. , Haramis, G. M. , Bunck, C. M. , & Pendleton, G. W. (1995). Effects of diet on rate of body mass gain by wintering canvasbacks. Journal of Wildlife Management, 59, 31–39.

[ece39905-bib-0078] Jourdan, C. , Fort, J. , Pinaud, D. , Delaporte, P. , Gernigon, J. , Guenneteau, S. , Jomat, L. , Lelong, V. , Lemesle, J.‐C. , Robin, F. , Rousseau, P. , & Bocher, P. (2021). Highly diversified habitats and resources influence habitat selection in wintering shorebirds. Journal of Ornithology, 162, 823–838.

[ece39905-bib-0079] Kang, S.‐R. , & King, S. L. (2014). Suitability of coastal marshes as whooping crane (*Grus americana*) foraging habitat in Southwest Louisiana, USA. Waterbirds, 37, 254–263.

[ece39905-bib-0080] Karlíková, Z. , Kejzlarová, T. , & Šálek, M. (2018). Breath rate patterns in precocial northern lapwing (*Vanellus vanellus*) chicks in the wild. Journal of Ornithology, 159, 555–563.

[ece39905-bib-0081] Kearney, M. (2006). Habitat, environment and niche: What are we modelling? Oikos, 115, 186–191.

[ece39905-bib-0082] Kelly, J. P. (2001). Hydrographic correlates of winter dunlin abundance and distribution in a temperate estuary. Waterbirds, 24, 309–322.

[ece39905-bib-0083] Kenow, K. P. , Kapfer, J. M. , & Korschgen, C. E. (2009). Predation of radio‐marked mallard (*Anas platyrhynchos*) ducklings by eastern snapping turtles (*Chelydra serpentina serpentina*) and Western Fox snakes (*Pantherophis vulpinus*) on the upper Mississippi River. Journal of Herpetology, 43, 154–158.

[ece39905-bib-0084] Khan, T. N. (2010). Temporal changes to the abundance and community structure of migratory waterbirds in Santragachhi Lake, West Bengal, and their relationship with water hyacinth cover. Current Science, 99, 1570–1577.

[ece39905-bib-0085] Klaassen, M. , Hahn, S. , Korthals, H. , & Madsen, J. (2017). Eggs brought in from afar: Svalbard‐breeding pink‐footed geese can fly their eggs across the Barents Sea. Journal of Avian Biology, 48, 173–179.

[ece39905-bib-0086] Knutson, M. G. , Powell, L. A. , Hines, R. K. , Friberg, M. A. , & Niemi, G. J. (2006). An assessment of bird habitat quality using population growth rates. Condor, 108, 301–314.

[ece39905-bib-0087] Koivula, K. , & Rönkä, A. (1998). Habitat deterioration and efficiency of antipredator strategy in a meadow‐breeding wader, Temminck's stint (*Calidris temminckii*). Oecologia, 116, 348–355. 28308066 10.1007/s004420050597

[ece39905-bib-0088] Kosztolányi, A. , Székely, T. , Cuthill, I. C. , Yilmaz, K. T. , & Berberoǧlu, S. (2006). Ecological constraints on breeding system evolution: The influence of habitat on brood desertion in Kentish plover. Journal of Animal Ecology, 75, 257–265. 16903063 10.1111/j.1365-2656.2006.01049.x

[ece39905-bib-0089] Krijgsveld, K. L. , Reneerkens, J. W. H. , Mcnett, G. D. , & Ricklefs, R. E. (2003). Time budgets and body temperatures of American Golden‐plover chicks in relation to ambient temperature. Condor, 105, 268–278.

[ece39905-bib-0090] Kruuk, L. E. B. , Clutton‐Brock, T. H. , Rose, K. E. , & Guinness, F. E. (1999). Early determinants of lifetime reproductive success differ between the sexes in Red Deer. Proceedings: Biological Sciences, 266, 1655–1661. 10501037 10.1098/rspb.1999.0828 PMC1690188

[ece39905-bib-0091] Kuwae, T. , Miyoshi, E. , Sassa, S. , & Watabe, Y. (2010). Foraging mode shift in varying environmental conditions by dunlin *Calidris alpina* . Marine Ecology Progress Series, 406, 281–289.

[ece39905-bib-0092] Kwon, E. , Robinson, S. , Weithman, C. E. , Catlin, D. H. , Karpanty, S. M. , Altman, J. , Simons, T. R. , & Fraser, J. D. (2021). Contrasting long‐term population trends of beach‐nesting shorebirds under shared environmental pressures. Biological Conservation, 260, 109178.

[ece39905-bib-0093] Lantz, S. M. , Gawlik, D. E. , & Cook, M. I. (2011). The effects of water depth and emergent vegetation on foraging success and habitat selection of wading birds in the Everglades. Waterbirds, 34, 439–447.

[ece39905-bib-0094] Le Boeuf, B. , Condit, R. , & Reiter, J. (2019). Lifetime reproductive success of northern elephant seals (*Mirounga angustirostris*). Canadian Journal of Zoology, 97, 1203–1217.

[ece39905-bib-0095] Le Fer, D. , Fraser, J. D. , & Kruse, C. D. (2008). Piping plover chick foraging, growth, and survival in the Great Plains. Journal of Wildlife Management, 72, 682–687.

[ece39905-bib-0096] Lebeuf, A. P. , & Giroux, J.‐F. (2014). Density‐dependent effects on nesting success of temperate‐breeding Canada geese. Journal of Avian Biology, 45, 600–608.

[ece39905-bib-0097] Lecomte, N. , Gauthier, G. , & Giroux, J. F. (2008). Breeding dispersal in a heterogeneous landscape: The influence of habitat and nesting success in greater snow geese. Oecologia, 155, 33–41. 17938972 10.1007/s00442-007-0860-6

[ece39905-bib-0098] Legagneux, P. , Harms, N. J. , Gauthier, G. , Chastel, O. , Gilchrist, H. G. , Bortolotti, G. , Bêty, J. , & Soos, C. (2013). Does feather corticosterone reflect individual quality or external stress in arctic‐nesting migratory birds? PLoS One, 8, e82644. 24391720 10.1371/journal.pone.0082644 PMC3877000

[ece39905-bib-0099] Lewis, T. , Flint, P. L. , Schmutz, J. A. , & Derksen, D. V. (2010). Pre‐moult patterns of habitat use and moult site selection by Brent geese *Branta bernicla nigricans*: Individuals prospect for moult sites. Ibis, 152, 556–568.

[ece39905-bib-0100] Li, X. , Hou, X. , Song, Y. , Shan, K. , Zhu, S. , Yu, X. , & Mo, X. (2019). Assessing changes of habitat quality for shorebirds in stopover sites: A case study in Yellow River Delta, China. Wetlands, 39, 67–77.

[ece39905-bib-0101] Liu, S.‐L. , Cai, Y.‐J. , Pang, S.‐L. , Qiu, F.‐C. , He, C.‐G. , & Song, Y.‐J. (2006). The influence of water resource condition on rare waterfowl in Zhalong National Nature Reserve. Journal of Northeast Normal University Natural Sciences Edition, 38, 105–108.

[ece39905-bib-0102] Loewenthal, D. , Paijmans, D. M. , & Hockey, P. A. R. (2015). Year‐round territoriality in long‐lived birds: Rethinking the concept of carrying capacity. Ostrich, 86, 23–34.

[ece39905-bib-0103] Lok, T. , Overdijk, O. , Tinbergen, J. M. , & Piersma, T. (2011). The paradox of spoonbill migration: Most birds travel to where survival rates are lowest. Animal Behaviour, 82, 837–844.

[ece39905-bib-0104] Lourenço, P. M. , Granadeiro, J. P. , & Palmeirim, J. M. (2005). Importance of drainage channels for waders foraging on tidal flats: Relevance for the management of estuarine wetlands. Journal of Applied Ecology, 42, 477–486.

[ece39905-bib-0105] Lovvorn, J. R. , North, C. A. , Grebmeier, J. M. , Cooper, L. W. , & Kolts, J. M. (2018). Sediment organic carbon integrates changing environmental conditions to predict benthic assemblages in shallow Arctic seas. Aquatic Conservation: Marine and Freshwater Ecosystems, 28, 861–871.

[ece39905-bib-0106] Lyons, J. E. , Collazo, J. A. , & Guglielmo, C. G. (2008). Plasma metabolites and migration physiology of semipalmated sandpipers: Refueling performance at five latitudes. Oecologia, 155, 417–427. 18071757 10.1007/s00442-007-0921-x

[ece39905-bib-0107] Ma, L. , Chen, C. , Shen, Y. , Wu, L.‐F. , Huang, Z.‐L. , & Cao, H.‐L. (2014). Determinants of tree survival at local scale in a sub‐tropical forest. Ecological Research, 29, 69–80.

[ece39905-bib-0108] Ma, Z. , Cai, Y. , Li, B. , & Chen, J. (2010). Managing wetland habitats for waterbirds: An international perspective. Wetlands, 30, 15–27.

[ece39905-bib-0109] Machín, P. , Fernández‐Elipe, J. , Hungar, J. , Angerbjörn, A. , Klaassen, R. H. G. , & Aguirre, J. I. (2019). The role of ecological and environmental conditions on the nesting success of waders in sub‐Arctic Sweden. Polar Biology, 42, 1571–1579.

[ece39905-bib-0110] Mallory, M. L. , Mcnicol, D. K. , & Weatherhead, P. J. (1994). Habitat quality and reproductive effort of common goldeneyes nesting near Sudbury, Canada. The Journal of Wildlife Management, 58, 552–560.

[ece39905-bib-0111] Mallory, M. L. , Walton, R. A. , & Mcnicol, D. K. (1999). Influence of intraspecific competition and habitat quality on diurnal activity budgets of breeding common goldeneyes. Écoscience, 6, 481–486.

[ece39905-bib-0112] Mander, L. , Marie‐Orleach, L. , & Elliott, M. (2013). The value of wader foraging behaviour study to assess the success of restored intertidal areas. Estuarine, Coastal and Shelf Science, 131, 1–5.

[ece39905-bib-0113] Manton, M. , & Angelstam, P. (2021). Macroecology of north european wet grassland landscapes: Habitat quality, waders, avian predators and nest predation. Sustainability (Switzerland), 13, 8138.

[ece39905-bib-0114] Markkola, J. A. , & Karvonen, R. T. (2020). Changing environmental conditions and structure of a breeding population of the threatened lesser white‐fronted goose (*Anser erythropus* L.). Ornis Fennica, 97, 113–130.

[ece39905-bib-0115] Marmillot, V. , Gauthier, G. , Cadieux, M. C. , & Legagneux, P. (2016). Plasticity in moult speed and timing in an arctic‐nesting goose species. Journal of Avian Biology, 47, 650–658.

[ece39905-bib-0116] Marzluff, J. M. , Raphael, M. G. , & Sallabanks, R. (2000). Understanding the effects of forest management on avian species. Wildlife Society Bulletin, 28, 1132–1143.

[ece39905-bib-0117] Mascitti, V. (2001). Habitat changes in Laguna de Pozuelos, Jujuy, Argentina: Implication for south American flamingo populations. Waterbirds, 24, 16–21.

[ece39905-bib-0118] Maslo, B. , Burger, J. , & Handel, S. N. (2012). Modeling foraging behavior of piping plovers to evaluate habitat restoration success. The Journal of Wildlife Management, 76, 181–188.

[ece39905-bib-0119] Master, T. L. , Leiser, J. K. , Bennett, K. A. , Bretsch, J. K. , & Wolfe, H. J. (2005). Patch selection by snowy egrets. Waterbirds, 28, 220–224.

[ece39905-bib-0120] Mattsson, B. J. , Runge, M. C. , Devries, J. H. , Boomer, G. S. , Eadie, J. M. , Haukos, D. A. , Fleskes, J. P. , Koons, D. N. , Thogmartin, W. E. , & Clark, R. G. (2012). A modeling framework for integrated harvest and habitat management of North American waterfowl: Case‐study of northern pintail metapopulation dynamics. Ecological Modelling, 225, 146–158.

[ece39905-bib-0121] McComb, B. C. (2016). Wildlife habitat management: Concepts and applications in forestry. CRC Press.

[ece39905-bib-0122] McKinney, R. A. , & McWilliams, S. R. (2005). A new model to estimate daily energy expenditure for wintering waterfowl. Wilson Bulletin, 117, 44–55.

[ece39905-bib-0123] McKinnon, L. , Schmaltz, L. , Aubry, Y. , Rochepault, Y. , Buidin, C. , & Juillet, C. (2022). Female migration phenology and climate conditions explain juvenile red knot (*Calidris canutus rufa*) counts during fall migration. Avian Conservation and Ecology, 17. 10.5751/ACE-02021-170109

[ece39905-bib-0124] Meltofte, H. (2006). Wader populations at Zackenberg, high‐arctic Northeast Greenland, 1996–2005. Dansk Ornitologisk Forenings Tidsskrift, 100, 16–28.

[ece39905-bib-0125] Merendino, M. T. , & Ankney, C. D. (1994). Habitat use by mallards and American black ducks breeding in Central Ontario. The Condor, 96, 411–421.

[ece39905-bib-0126] Merendino, M. T. , Dennis, D. G. , & Ankney, C. D. (1992). Mallard harvest data: An index of wetland quality for breeding waterfowl. Wildlife Society Bulletin, 20, 171–175.

[ece39905-bib-0127] Merrill, L. , Levengood, J. M. , England, J. C. , Osborn, J. M. , & Hagy, H. M. (2018). Blood parasite infection linked to condition of spring‐migrating lesser Scaup (*Aythya affinis*). Canadian Journal of Zoology, 96, 1145–1152.

[ece39905-bib-0128] Meyer, N. , Bollache, L. , Galipaud, M. , Moreau, J. , Dechaume‐Moncharmont, F. X. , Afonso, E. , Angerbjörn, A. , Bêty, J. , Brown, G. , Ehrich, D. , Gilg, V. , Giroux, M. A. , Hansen, J. , Lanctot, R. , Lang, J. , Latty, C. , Lecomte, N. , McKinnon, L. , Kennedy, L. , … Gilg, O. (2021). Behavioural responses of breeding arctic sandpipers to ground‐surface temperature and primary productivity. Science of the Total Environment, 755, 142485. 33039934 10.1016/j.scitotenv.2020.142485

[ece39905-bib-0129] Milenkaya, O. , Catlin, D. H. , Legge, S. , & Walters, J. R. (2015). Body condition indices predict reproductive success but not survival in a sedentary, tropical bird. PLoS ONE, 10, e0136582. 26305457 10.1371/journal.pone.0136582 PMC4549336

[ece39905-bib-0130] Minias, P. , & Janiszewski, T. (2016). Territory selection in the city: Can birds reliably judge territory quality in a novel urban environment? Journal of Zoology, 300, 120–126.

[ece39905-bib-0131] Mokany, K. , Ware, C. , Harwood, T. D. , Schmidt, R. K. , & Ferrier, S. (2022). Habitat‐based biodiversity assessment for ecosystem accounting in the Murray–Darling Basin. Conservation Biology, 36, e13915. 35384070 10.1111/cobi.13915 PMC9796243

[ece39905-bib-0132] Moloney, P. D. , Gormley, A. M. , Toop, S. D. , Flesch, J. S. , Forsyth, D. M. , Ramsey, D. S. L. , & Hampton, J. O. (2022). Bayesian modelling reveals differences in long‐term trends in the harvest of native and introduced species by recreational hunters in Australia. Wildlife Research, 49, 673–685. 10.1071/WR21138

[ece39905-bib-0133] Mu, T. , Cai, S. , Peng, H. B. , Hassell, C. J. , Boyle, A. , Zhang, Z. , Piersma, T. , & Wilcove, D. S. (2022). Evaluating staging habitat quality to advance the conservation of a declining migratory shorebird, red knot *Calidris canutus* . Journal of Applied Ecology, 59, 2084–2093.

[ece39905-bib-0134] Murray, D. L. , Anderson, M. G. , & Steury, T. D. (2010). Temporal shift in density dependence among North American breeding duck populations. Ecology, 91, 571–581. 20392021 10.1890/ms08-1032.1

[ece39905-bib-0135] Nam, H. K. , Choi, Y. S. , Choi, S. H. , & Yoo, J. C. (2015). Distribution of waterbirds in Rice fields and their use of foraging habitats. Waterbirds, 38, 173–183.

[ece39905-bib-0136] Neuman, K. K. , Henkel, L. A. , & Page, G. W. (2008). Shorebird use of sandy beaches in Central California. Waterbirds, 31, 115–121.

[ece39905-bib-0137] Nolet, B. A. , De Boer, T. , & De Vries, P. P. (2007). Habitat quality estimated from head‐dipping time in trampling swans. Israel Journal of Ecology and Evolution, 53, 317–328.

[ece39905-bib-0138] Nolet, B. A. , & Klaassen, M. (2009). Retrodicting patch use by foraging swans in a heterogeneous environment using a set of functional responses. Oikos, 118, 431–439.

[ece39905-bib-0139] Ntiamoa‐Baidu, Y. , Nuoh, A. A. , Reneerkens, J. , & Piersma, T. (2014). Population increases in non‐breeding sanderlings in Ghana indicate site preference. Ardea, 102, 131–137.

[ece39905-bib-0140] Nyman, J. A. , & Chabreck, R. H. (1996). Some effects of 30 years of weir‐management on coastal marsh aquatic vegetation and implications to waterfowl management. Gulf of Mexico Science, 14, 16–25.

[ece39905-bib-0141] O'Neal, B. J. , Stafford, J. D. , & Larkin, R. P. (2012). Stopover duration of fall‐migrating dabbling ducks. The Journal of Wildlife Management, 76, 285–293.

[ece39905-bib-0142] O'Neil, S. T. , Warren, J. M. , Takekawa, J. Y. , De La Cruz, S. E. W. , Cutting, K. A. , Parker, M. W. , & Yee, J. L. (2014). Behavioural cues surpass habitat factors in explaining prebreeding resource selection by a migratory diving duck. Animal Behaviour, 90, 21–29.

[ece39905-bib-0143] Osborn, J. M. , Hagy, H. M. , Mcclanahan, M. D. , & Gray, M. J. (2017). Temporally robust models for predicting seed yield of moist‐soil plants. Wildlife Society Bulletin, 41, 157–161.

[ece39905-bib-0144] Owen, T. M. , & Pierce, A. R. (2014). Productivity and chick growth rates of royal tern (*Thalasseus maximus*) and sandwich tern (*Thalasseus sandvicensis*) on the isles Dernieres Barrier Island refuge, Louisiana. Waterbirds, 37, 245–253.

[ece39905-bib-0145] Parks, M. A. , Collazo, J. A. , Colón, J. A. , Álvarez, K. R. R. , & Díaz, O. (2016). Change in numbers of resident and migratory shorebirds at the Cabo Rojo salt flats, Puerto Rico, USA (1985–2014). Waterbirds, 39, 209–214.

[ece39905-bib-0146] Pedler, R. D. , Ribot, R. F. H. , & Bennett, A. T. D. (2014). Extreme nomadism in desert waterbirds: Flights of the banded stilt. Biology Letters, 10, 20140547. 25319819 10.1098/rsbl.2014.0547 PMC4272204

[ece39905-bib-0147] Pehlak, H. , & Lõhmus, A. (2008). An artificial nest experiment indicates equal nesting success of waders in coastal meadows and mires. Ornis Fennica, 85, 66–71.

[ece39905-bib-0148] Pérot, A. , & Villard, M.‐A. (2009). Putting density back into the habitat‐quality equation: Case study of an open‐nesting forest bird. Conservation Biology, 23, 1550–1557. 19558524 10.1111/j.1523-1739.2009.01272.x

[ece39905-bib-0149] Peters, K. A. , & Otis, D. L. (2007). Shorebird roost‐site selection at two temporal scales: Is human disturbance a factor? Journal of Applied Ecology, 44, 196–209.

[ece39905-bib-0150] Pezzanite, B. , Rockwell, R. F. , Davies, J. C. , Loonen, M. J. J. E. , & Seguin, R. J. (2005). Has habitat degradation affected foraging behaviour and reproductive success of lesser snow geese (Chen caerulescens caerulescens)? Ecoscience, 12, 439–446.

[ece39905-bib-0151] Pidgeon, A. M. , Radeloff, V. C. , & Mathews, N. E. (2006). Contrasting measures of fitness to classify habitat quality for the black‐throated sparrow (*Amphispiza bilineata*). Biological Conservation, 132, 199–210.

[ece39905-bib-0152] Pierce, A. K. , Dinsmore, S. J. , & Wunder, M. B. (2019). Decreased nest survival associated with low temperatures in a high‐elevation population of mountain plover (*Charadrius montanus*). Wilson Journal of Ornithology, 131, 502–513.

[ece39905-bib-0153] Piersma, T. , Lok, T. , Chen, Y. , Hassell, C. J. , Yang, H.‐Y. , Boyle, A. , Slaymaker, M. , Chan, Y.‐C. , Melville, D. S. , Zhang, Z.‐W. , & Ma, Z. (2016). Simultaneous declines in summer survival of three shorebird species signals a flyway at risk. Journal of Applied Ecology, 53, 479–490.

[ece39905-bib-0154] Powell, G. V. N. , & Powell, A. H. (1986). Reproduction by great white herons *Ardea herodias* in Florida bay as an indicator of habitat quality. Biological Conservation, 36, 101–113.

[ece39905-bib-0155] Pöysä, H. , Sjöberg, K. , Elmberg, J. , & Nummi, P. (2001). Pair formation among experimentally introduced mallards *Anas platyrhynchos* reflects habitat quality. Annales Zoologici Fennici, 38, 179–184.

[ece39905-bib-0156] Pulliam, H. R. (2000). On the relationship between niche and distribution. Ecology Letters, 3, 349–361.

[ece39905-bib-0157] Pullin, A. S. , & Stewart, G. B. (2006). Guidelines for systematic review in conservation and environmental management. Conservation Biology, 20, 1647–1656. 17181800 10.1111/j.1523-1739.2006.00485.x

[ece39905-bib-0158] Quan, R.‐C. , Wen, X. , & Yang, X. (2002). Effects of human activities on migratory waterbirds at Lashihai Lake, China. Biological Conservation, 108, 273–279.

[ece39905-bib-0159] Raquel, A. J. , Devries, J. H. , Howerter, D. W. , Alisauskas, R. T. , Leach, S. W. , & Clark, R. G. (2016). Timing of nesting of upland‐nesting ducks in the Canadian Prairies and its relation to spring wetland conditions. Canadian Journal of Zoology, 94, 575–581.

[ece39905-bib-0160] Reed, E. T. , Gauthier, G. , Pradel, R. , & Lebreton, J. D. (2003). Age and environmental conditions affect recruitment in greater snow geese. Ecology, 84, 219–230.

[ece39905-bib-0161] Reurink, F. , Hentze, N. , Rourke, J. , & Ydenberg, R. (2015). Site‐specific flight speeds of nonbreeding Pacific dunlins as a measure of the quality of a foraging habitat. Behavioral Ecology, 27, 803–809.

[ece39905-bib-0162] Reynolds, M. H. , Hatfield, J. S. , Laniawe, L. P. , Vekasy, M. S. , Klavitter, J. L. , Berkowitz, P. , Crampton, L. H. , & Walters, J. R. (2012). Influence of space use on fitness and the reintroduction success of the Laysan teal. Animal Conservation, 15, 305–317.

[ece39905-bib-0163] Rice, S. M. , Collazo, J. A. , Alldredge, M. W. , Harrington, B. A. , & Lewis, A. R. (2007). Local annual survival and seasonal residency rates of semipalmated sandpipers (*Calidris pusilla*) in Puerto Rico. The Auk, 124, 1397–1406.

[ece39905-bib-0164] Richard, E. , Simpson, S. E. , Medill, S. A. , & Mcloughlin, P. D. (2014). Interacting effects of age, density, and weather on survival and current reproduction for a large mammal. Ecology and Evolution, 4, 3851–3860. 25614799 10.1002/ece3.1250 PMC4301048

[ece39905-bib-0165] Riecke, T. V. , Conway, W. C. , Haukos, D. A. , Moon, J. A. , & Comer, C. E. (2019). Nest survival of black‐necked stilts (*Himantopus mexicanus*) on the upper Texas coast, USA. Waterbirds, 42, 261–271.

[ece39905-bib-0166] Ringelman, K. M. , Walker, J. , Ringelman, J. K. , & Stephens, S. E. (2018). Temporal and multi‐spatial environmental drivers of duck nest survival. Auk, 135, 486–494.

[ece39905-bib-0167] Rizzolo, D. J. , Schmutz, J. A. , & Speakman, J. R. (2015). Fast and efficient: Postnatal growth and energy expenditure in an Arctic‐breeding waterbird, the red‐throated loon (*Gavia stellata*). Auk, 132, 657–670.

[ece39905-bib-0168] Rogers, K. G. , & Gosbell, K. (2006). Demographic models for red‐necked stint and curlew sandpiper in Victoria. Stilt, 50, 205–214.

[ece39905-bib-0169] Rose, M. , & Nol, E. (2010). Foraging behavior of non‐breeding Semipalmated plovers. Waterbirds, 33, 59–69.

[ece39905-bib-0170] Roshier, D. , Klomp, N. , & Asmus, M. (2006). Movements of a nomadic waterfowl, grey teal *Anas gracilis*, across inland Australia–results from satellite telemetry spanning fifteen months. Ardea, 94, 461–475.

[ece39905-bib-0171] Ross, B. E. , Haukos, D. A. , & Walther, P. (2018). Quantifying changes and influences on mottled duck density in Texas. Journal of Wildlife Management, 82, 374–382.

[ece39905-bib-0172] Roy, C. L. , Berdeen, J. B. , & Clark, M. (2019). A demographic model for ring‐necked ducks breeding in Minnesota. Journal of Wildlife Management, 83, 1720–1734.

[ece39905-bib-0173] Russell, I. A. , Randall, R. M. , & Hanekom, N. (2014). Spatial and temporal patterns of waterbird assemblages in the wilderness lakes complex, South Africa. Waterbirds, 37, 1–18.

[ece39905-bib-0174] Ruthrauff, D. R. , Dekinga, A. , Gill, R. E., Jr. , Summers, R. W. , & Piersma, T. (2013). Ecological correlates of variable organ sizes and fat loads in the most northerly wintering shorebirds. Canadian Journal of Zoology, 91, 698–705.

[ece39905-bib-0175] Sabatier, R. , Doyen, L. , & Tichit, M. (2010). Modelling trade‐offs between livestock grazing and wader conservation in a grassland agroecosystem. Ecological Modelling, 221, 1292–1300.

[ece39905-bib-0176] Saunders, S. P. , Piper, W. , Farr, M. T. , Bateman, B. L. , Michel, N. L. , Westerkam, H. , & Wilsey, C. B. (2021). Interrelated impacts of climate and land‐use change on a widespread waterbird. Journal of Animal Ecology, 90, 1165–1176. 33754380 10.1111/1365-2656.13444

[ece39905-bib-0177] Schaffer‐Smith, D. , Swenson, J. J. , Reiter, M. E. , & Isola, J. E. (2018). Quantifying shorebird habitat in managed wetlands by modeling shallow water depth dynamics. Ecological Applications, 28, 1534–1545. 29694689 10.1002/eap.1732 PMC6123259

[ece39905-bib-0178] Schlägel, U. E. , & Mädlow, W. (2022). All‐season space use by non‐native resident mandarin ducks (*Aix galericulata*) in northeastern Germany. Journal of Ornithology, 163, 71–82.

[ece39905-bib-0179] Schultz, R. , Straub, J. , Kaminski, M. , & Ebert, A. (2020). Floristic and macroinvertebrate responses to different wetland restoration techniques in southeastern Wisconsin. Wetlands, 40, 2025–2040.

[ece39905-bib-0180] Schummer, M. L. , Kaminski, R. M. , Raedeke, A. H. , & Graber, D. A. (2010). Weather‐related indices of autumn–winter dabbling duck abundance in middle North America. Journal of Wildlife Management, 74, 94–101.

[ece39905-bib-0181] Sebastián‐González, E. , Botella, F. , Sempere, R. A. , & Sánchez‐Zapata, J. A. (2010). An empirical demonstration of the ideal free distribution: Little grebes *Tachybaptus ruficollis* breeding in intensive agricultural landscapes. Ibis, 152, 643–650.

[ece39905-bib-0182] Sebastián‐González, E. , Sánchez‐Zapata, J. A. , & Botella, F. (2010). Agricultural ponds as alternative habitat for waterbirds: Spatial and temporal patterns of abundance and management strategies. European Journal of Wildlife Research, 56, 11–20.

[ece39905-bib-0183] Sebastián‐González, E. , Sánchez‐Zapata, J. A. , Botella, F. , & Otso, O. (2010). Testing the heterospecific attraction hypothesis with time‐series data on species co‐occurrence. Proceedings of the Royal Society B: Biological Sciences, 277, 2983–2990. 10.1098/rspb.2010.0244 PMC2982017 20462909

[ece39905-bib-0184] Sedinger, J. S. , & Alisauskas, R. T. (2014). Cross‐seasonal effects and the dynamics of waterfowl populations. Wild, 4, 277–304.

[ece39905-bib-0185] Simpson, J. W. , Yerkes, T. , Nudds, T. D. , & Smith, B. D. (2007). Effects of habitat on mallard duckling survival in the Great Lakes region. Journal of Wildlife Management, 71, 1885–1891.

[ece39905-bib-0186] Sjöberg, K. , Pöysä, H. , Elmberg, J. , & Nummi, P. (2000). Response of mallard ducklings to variation in habitat quality: An experiment of food limitation. Ecology, 81, 329–335.

[ece39905-bib-0187] Smart, J. , Bolton, M. , Hunter, F. , Quayle, H. , Thomas, G. , & Gregory, R. D. (2013). Managing uplands for biodiversity: Do Agri‐environment schemes deliver benefits for breeding lapwing Vanellus vanellus? Journal of Applied Ecology, 50, 794–804.

[ece39905-bib-0188] Smart, J. , & Gill, J. A. (2003). Non‐intertidal habitat use by shorebirds: A reflection of inadequate intertidal resources? Biological Conservation, 111, 359–369.

[ece39905-bib-0189] Smith, J. P. (1995). Foraging sociability of nesting wading birds (Ciconiiformes) at Lake Okeechobee, Florida. The Wilson Bulletin, 107, 437–451.

[ece39905-bib-0190] Sorenson, L. G. , Goldberg, R. , Root, T. L. , & Anderson, M. G. (1998). Potential effects of global warming on waterfowl populations breeding in the northern Great Plains. Climatic Change, 40, 343–369.

[ece39905-bib-0191] Souchay, G. , Gauthier, G. , Lefebvre, J. , & Pradel, R. (2015). Absence of difference in survival between two distant breeding sites of greater snow geese. Journal of Wildlife Management, 79, 570–578.

[ece39905-bib-0192] Steen, V. , Skagen, S. K. , & Noon, B. R. (2018). Preparing for an uncertain future: Migrating shorebird response to past climatic fluctuations in the prairie potholes: Migrating. Ecosphere, 9, e02095.

[ece39905-bib-0193] Stephens, P. A. , Pettorelli, N. , Barlow, J. , Whittingham, M. J. , & Cadotte, M. W. (2015). Management by proxy? The use of indices in applied ecology. Journal of Applied Ecology, 52, 1–6.

[ece39905-bib-0194] Stillman, R. A. , Goss‐Custard, J. D. , West, A. D. , Dit Durell, S. E. L. V. , Caldow, R. W. G. , Mcgrorty, S. , & Clarke, R. T. (2000). Predicting mortality in novel environments: Tests and sensitivity of a behaviour‐based model. Journal of Applied Ecology, 37, 564–588.

[ece39905-bib-0195] Stillman, R. A. , West, A. D. , Goss‐Custard, J. D. , Mcgrorty, S. , Frost, N. J. , Morrisey, D. J. , Kenny, A. J. , & Drewitt, A. L. (2005). Predicting site quality for shorebird communities: A case study on the Humber estuary, UK. Marine Ecology Progress Series, 305, 203–217.

[ece39905-bib-0196] Stolen, E. D. , Collazo, J. A. , & Percival, H. F. (2012). Group‐foraging effects on capture rate in wading birds. The Condor, 114, 744–754.

[ece39905-bib-0197] Studds, C. E. , Kendall, B. E. , Murray, N. J. , Wilson, H. B. , Rogers, D. I. , Clemens, R. S. , Gosbell, K. , Hassell, C. J. , Jessop, R. , Melville, D. S. , Milton, D. A. , Minton, C. D. T. , Possingham, H. P. , Riegen, A. C. , Straw, P. , Woehler, E. J. , & Fuller, R. A. (2017). Rapid population decline in migratory shorebirds relying on Yellow Sea tidal mudflats as stopover sites. Nature Communications, 8, 14895. 10.1038/ncomms14895 PMC5399291 28406155

[ece39905-bib-0198] Swift, R. J. , Rodewald, A. D. , Johnson, J. A. , Andres, B. A. , & Senner, N. R. (2020). Seasonal survival and reversible state effects in a long‐distance migratory shorebird. Journal of Animal Ecology, 89, 2043–2055. 32358801 10.1111/1365-2656.13246

[ece39905-bib-0199] Tang, X. , Li, H. , Xu, X. , Yang, G. , Liu, G. , Li, X. , & Chen, D. (2016). Changing land use and its impact on the habitat suitability for wintering Anseriformes in China's Poyang Lake region. Science of the Total Environment, 557–558, 296–306. 10.1016/j.scitotenv.2016.03.108 27016677

[ece39905-bib-0200] Tavernia, B. G. , & Reed, J. M. (2012). The impact of exotic purple loosestrife (*Lythrum salicaria*) on wetland bird abundances. The American Midland Naturalist, 168, 352–363.

[ece39905-bib-0201] Thibault, J. M. , Sanders, F. J. , & Jodice, P. G. R. (2010). Parental attendance and brood success in American oystercatchers in South Carolina. Waterbirds, 33, 511–517.

[ece39905-bib-0202] Thomas, N. E. , & Swanson, D. L. (2013). Plasma metabolites and creatine kinase levels of shorebirds during fall migration in the prairie pothole region. The Auk, 130, 580–590.

[ece39905-bib-0203] Tombre, I. M. , Tømmervik, H. , & Madsen, J. (2005). Land use changes and goose habitats, assessed by remote sensing techniques, and corresponding goose distribution, in Vesterålen, northern Norway. Agriculture, Ecosystems & Environment, 109, 284–296.

[ece39905-bib-0204] Toral, G. M. , Stillman, R. A. , Santoro, S. , & Figuerola, J. (2012). The importance of rice fields for glossy ibis (*Plegadis falcinellus*): Management recommendations derived from an individual‐based model. Biological Conservation, 148, 19–27.

[ece39905-bib-0205] Townsend, S. , Pearlstine, E. , Mazzotti, F. , & Deren, C. (2006). Wading birds, shorebirds, and waterfowl in rice fields within the Everglades agricultural area. Florida Field Naturalist, 34, 9–20.

[ece39905-bib-0206] Trinder, M. N. , Hassell, D. , & Votier, S. (2009). Reproductive performance in arctic‐nesting geese is influenced by environmental conditions during the wintering, breeding and migration seasons. Oikos, 118, 1093–1101.

[ece39905-bib-0207] Valdez‐Juarez, S. O. , Krebs, E. A. , Drake, A. E. , & Green, D. J. (2019). Assessing the effect of seasonal agriculture on the condition and winter survival of a migratory songbird in Mexico. Conservation Science and Practice, 1, e19.

[ece39905-bib-0208] van de Pol, M. , Brouwer, L. , Ens, B. J. , Oosterbeek, K. , & Tinbergen, J. M. (2010). Fluctuating selection and the maintenance of individual and sex‐specific diet specialization in free‐living oystercatchers. Evolution, 64, 836–851. 19804401 10.1111/j.1558-5646.2009.00859.x

[ece39905-bib-0209] van de Pol, M. , Ens, B. J. , Heg, D. , Brouwer, L. , Krol, J. , Maier, M. , Exo, K. M. , Oosterbeek, K. , Lok, T. , Eising, C. M. , & Koffijberg, K. (2010). Do changes in the frequency, magnitude and timing of extreme climatic events threaten the population viability of coastal birds? Journal of Applied Ecology, 47, 720–730.

[ece39905-bib-0210] van der Kolk, H. J. , Ens, B. J. , Oosterbeek, K. , Bouten, W. , Allen, A. M. , Frauendorf, M. , Lameris, T. K. , Oosterbeek, T. , Deuzeman, S. , De Vries, K. , Jongejans, E. , & van de Pol, M. (2019). Shorebird feeding specialists differ in how environmental conditions alter their foraging time. Behavioral Ecology, 31, 371–382.

[ece39905-bib-0211] Van Gils, J. A. , Dekinga, A. , Spaans, B. , Vahl, W. K. , & Piersma, T. (2005). Digestive bottleneck affects foraging decisions in red knots *Calidris canutus*. II. Patch choice and length of working day. Journal of Animal Ecology, 74, 120–130.

[ece39905-bib-0212] Van Horne, B. (1983). Density as a misleading indicator of habitat quality. The Journal of Wildlife Management, 47, 893–901.

[ece39905-bib-0213] Walsh, K. A. , Halliwell, D. R. , Hines, J. E. , Fournier, M. A. , Czarnecki, A. , & Dahl, M. F. (2006). Effects of water quality on habitat use by lesser scaup (*Aythya affinis*) broods in the boreal Northwest Territories, Canada. Hydrobiologia, 567, 101–111.

[ece39905-bib-0214] Wang, T. , & So, S. (2003). Waterbirds spending the boreal summer at Dalai Nur National Nature Reserve, Inner Mongolia, China. Bulletin of the Oriental Bird Club, 38, 58–61.

[ece39905-bib-0215] Wang, X. , Comita, L. S. , Hao, Z. , Davies, S. J. , Ye, J. , Lin, F. , & Yuan, Z. (2012). Local‐scale drivers of tree survival in a temperate forest. PLoS One, 7, e29469. 22347996 10.1371/journal.pone.0029469 PMC3278403

[ece39905-bib-0216] Warnock, N. , Haig, S. M. , & Oring, L. W. (1998). Monitoring species richness and abundance of shorebirds in the western Great Basin. Condor, 100, 589–600.

[ece39905-bib-0217] Weegman, M. D. , Alisauskas, R. T. , Kellett, D. K. , Zhao, Q. , Wilson, S. , & Telenský, T. (2022). Local population collapse of Ross's and lesser snow geese driven by failing recruitment and diminished philopatry. Oikos, 2022, 1–13.

[ece39905-bib-0218] Weiser, E. L. , Brown, S. C. , Lanctot, R. B. , Gates, H. R. , Abraham, K. F. , Bentzen, R. L. , Bêty, J. , Boldenow, M. L. , Brook, R. W. , Donnelly, T. F. , English, W. B. , Flemming, S. A. , Franks, S. E. , Gilchrist, H. G. , Giroux, M.‐A. , Johnson, A. , Kendall, S. , Kennedy, L. V. , Koloski, L. , … Sandercock, B. K. (2018a). Effects of environmental conditions on reproductive effort and nest success of Arctic‐breeding shorebirds. Ibis, 160, 608–623.

[ece39905-bib-0219] Weiser, E. L. , Brown, S. C. , Lanctot, R. B. , Gates, H. R. , Abraham, K. F. , Bentzen, R. L. , Bêty, J. , Boldenow, M. L. , Brook, R. W. , Donnelly, T. F. , English, W. B. , Flemming, S. A. , Franks, S. E. , Gilchrist, H. G. , Giroux, M. A. , Johnson, A. , Kennedy, L. V. , Koloski, L. , Kwon, E. , … Sandercock, B. K. (2018b). Life‐history tradeoffs revealed by seasonal declines in reproductive traits of Arctic‐breeding shorebirds. Journal of Avian Biology, 49, jav.01531.

[ece39905-bib-0220] Wen, L. , Saintilan, N. , Reid, J. R. W. , & Colloff, M. J. (2016). Changes in distribution of waterbirds following prolonged drought reflect habitat availability in coastal and inland regions. Ecology and Evolution, 6, 6672–6689. 27777739 10.1002/ece3.2091 PMC5058537

[ece39905-bib-0221] West, A. D. , Goss‐Custard, J. D. , Dit Durell, S. E. L. V. , & Stillman, R. A. (2005). Maintaining estuary quality for shorebirds: Towards simple guidelines. Biological Conservation, 123, 211–224.

[ece39905-bib-0222] Wetlands International . (2012). Waterbird population estimates, fifth edition. Summary report. Wetlands International.

[ece39905-bib-0223] Wiersma, P. , & Piersma, T. (1995). Scoring abdominal profiles to characterize migratory cohorts of shorebirds: An example with red knots. Journal of Field Ornithology, 66, 88–98.

[ece39905-bib-0224] Williams, B. R. , Benson, T. J. , Yetter, A. P. , Lancaster, J. D. , & Hagy, H. M. (2019). Stopover duration of spring migrating dabbling ducks in the Wabash River valley. Wildlife Society Bulletin, 43, 590–598.

[ece39905-bib-0225] Xu, F. , & Si, Y. (2019). The frost wave hypothesis: How the environment drives autumn departure of migratory waterfowl. Ecological Indicators, 101, 1018–1025.

[ece39905-bib-0226] Xu, Y. , Si, Y. , Yin, S. , Zhang, W. , Grishchenko, M. , Prins, H. H. T. , Gong, P. , & De Boer, W. F. (2019). Species‐dependent effects of habitat degradation in relation to seasonal distribution of migratory waterfowl in the East Asian–Australasian Flyway. Landscape Ecology, 34, 243–257.

[ece39905-bib-0227] Yu, C. , Zhou, L. , Mahtab, N. , Fan, S. , & Song, Y. (2020). Microhabitat variables explain patch switching by wintering Bewick's swans through giving‐up net energy intake rates. Environmental Science and Pollution Research, 27, 18843–18852. 32207014 10.1007/s11356-020-08343-w

[ece39905-bib-0228] Zanette, L. (2001). Indicators of habitat quality and the reproductive output of a forest songbird in small and large fragments. Journal of Avian Biology, 32, 38–46.

[ece39905-bib-0229] Zhang, W. , Li, X. , Yu, L. , & Si, Y. (2018). Multi‐scale habitat selection by two declining east Asian waterfowl species at their core spring stopover area. Ecological Indicators, 87, 127–135.

[ece39905-bib-0230] Zhang, Y. , Wang, Z. , Ren, C. , Yu, H. , Dong, Z. , Lu, C. , & Mao, D. (2017). Changes in habitat suitability for waterbirds of the Momoge nature Reserve of China during 1990–2014. Journal of Environmental Engineering and Landscape Management, 25, 367–378.

[ece39905-bib-0231] Zhao, D. , Borders, B. , Wilson, M. , & Rathbun, S. L. (2006). Modeling neighborhood effects on the growth and survival of individual trees in a natural temperate species‐rich forest. Ecological Modelling, 196, 90–102.

[ece39905-bib-0232] Zharikov, Y. , Elner, R. W. , Shepherd, P. C. F. , & Lank, D. B. (2009). Interplay between physical and predator landscapes affects transferability of shorebird distribution models. Landscape Ecology, 24, 129–144.

